# GMoE-AD: Generalized Hyperspectral Anomaly Detection via Mixture-of-Experts and Domain-Invariant Learning

**DOI:** 10.3390/s26175661

**Published:** 2026-09-06

**Authors:** Mazharul Hossain, Aaron Robinson, Chrysanthe Preza, Lan Wang

**Affiliations:** 1 Computer Science Department, The University of Memphis, Memphis, TN 38152, USA; 2 Electrical and Computer Engineering Department, The University of Memphis, Memphis, TN 38152, USA; alrobins@memphis.edu (A.R.); cpreza@memphis.edu (C.P.)

**Keywords:** hyperspectral anomaly detection, cross-domain learning, mixture of experts, domain adaptation, domain generalization, transfer learning

## Abstract

Hyperspectral (HS) sensing provides detailed high-dimensional spectral data to identify subtle anomalies and material variations in complex scenes. HS anomaly detection (HS-AD) aims to identify small, spectrally distinct objects or materials in HS imagery without prior target information. However, most HS-AD methods assume that the training and test data are drawn from the same distribution (single-domain). This assumption is often violated in operational sensing because of differences in sensor characteristics, scene composition, atmospheric conditions, and acquisition geometry. To address this challenge, we propose Generalized MoE-AD (GMoE-AD), a neural Mixture-of-Experts (MoE) architecture for robust cross-domain hyperspectral anomaly detection. The framework fuses the outputs of six unsupervised base detectors with representations from a pretrained HS foundation model, combines four neural experts through learned top-2 routing, and applies gradient-reversal-based domain-adversarial training to improve robustness under distribution shifts. A drift-aware test-time adaptation (DTA) variant is evaluated separately. We evaluate GMoE-AD on six public real-world HS benchmark datasets—San-Diego, Salinas, HYDICE-Urban, ABU-Airport, ABU-Beach, and ABU-Urban—and one private Arizona dataset comprising 22 images acquired by four different sensors. Under all-domain training, the model uses the training portions of all seven datasets and is evaluated without access to domain identity or dataset-specific information. GMoE-AD achieves an average ROC-AUC of 0.943, PR-AUC of 0.623, and F1-macro of 0.821 in this setting. In a leave-one-dataset-out (LODO) evaluation, where the target dataset is completely unseen during training, the model maintains an average ROC-AUC of 0.910 and F1-macro of 0.773. The optional DTA variant increases mean ROC-AUC from 0.938 to 0.953 but reduces F1-macro from 0.822 to 0.815, indicating a metric- and dataset-dependent adaptation trade-off. These results suggest that combining neural expert routing, transfer learning, and domain-adversarial representation learning improves robustness across heterogeneous HS datasets and provides a unified approach to anomaly detection in high-dimensional sensor data, supporting deployment in real-world hyperspectral applications where training and deployment conditions differ.

## 1. Introduction

Hyperspectral (HS) sensing provides dense spectral measurements that enable fine-grained material discrimination and reliable detection of subtle anomalies in complex scenes [1,2,3,4,5]. As a result, HS anomaly detection (HS-AD) has become an essential capability in applications such as environmental monitoring, precision agriculture, industrial inspection, and military reconnaissance [6,7,8,9,10].

Despite extensive progress, most HS-AD methods implicitly assume that training and test data are drawn from the same distribution. In practice, this assumption is often violated due to variations in scene content, atmospheric variability, sensor characteristics, and flight parameters. Such factors introduce significant spectral and statistical shifts across datasets, which can severely degrade detection performance [11,12,13]. This sensitivity to distributional variability motivates the development of more robust HS-AD frameworks that can generalize across the diverse operating conditions encountered by HS sensors [14]. While recent HS anomaly detection methods have focused on improving detection accuracy within individual datasets, most approaches are developed and evaluated under single-dataset settings with implicit distributional assumptions. In contrast, our work explicitly targets cross-domain generalization by training a unified model across multiple heterogeneous datasets and evaluating it without access to domain identity at inference time. Therefore, the proposed setting does not assume that training and deployment data share the same distribution; rather, it is designed for the practical case in which deployment data may originate from an unseen or shifted domain.

In our prior work, we introduced a stacking ensemble approach that combines HS unmixing methods [2] with unsupervised HS-AD algorithms [15], integrated through a supervised meta-classifier [16]. While these ensembles improved detection robustness, their performance still degraded under significant domain shifts between training and deployment data.

In this work, we approximate domains as dataset-specific distributions during training and treat each dataset as a separate domain, even when captured by the same sensor, due to variations in scene composition and anomaly features. While recognizing that true domain boundaries are more complex and may arise from factors such as sensor characteristics, acquisition conditions, and scene composition, datasets may exhibit overlapping or mixed domain characteristics rather than forming strictly isolated domains. In practical settings, explicit domain labels (e.g., sensor type or acquisition conditions) might be unavailable or unreliable during inference. Therefore, models need to infer latent domain characteristics directly from input data and route predictions accordingly. This motivates adaptive architectures that dynamically select and combine multiple detection strategies based on input features, enabling soft input-adaptive neural-expert weighting without relying on fixed dataset-specific models or explicit domain information during testing. Based on this motivation, our research hypothesis is that combining transferable foundation-model features with neural-expert routing can preserve useful domain-sensitive anomaly cues while improving robustness to cross-domain distribution shifts.

Recent HS-AD studies have improved within-dataset representation and reconstruction through deep plug-and-play denoising regularization, Transformer architectures, masked reconstruction, Mamba-based multiscale reconstruction, and self-supervised background modeling [13,17,18,19,20]. Separately, transfer-learning, cross-domain, and continual-learning methods highlight the importance of robustness when hyperspectral scenes change over time or across acquisition domains [21,22,23,24]. These lines of work generally focus on individual datasets, predefined source–target settings, or sequential adaptation. This work instead studies a unified cross-domain HS-AD setting in which one model is trained using all available training datasets and used at inference without dataset identity.

**Figure 1:**
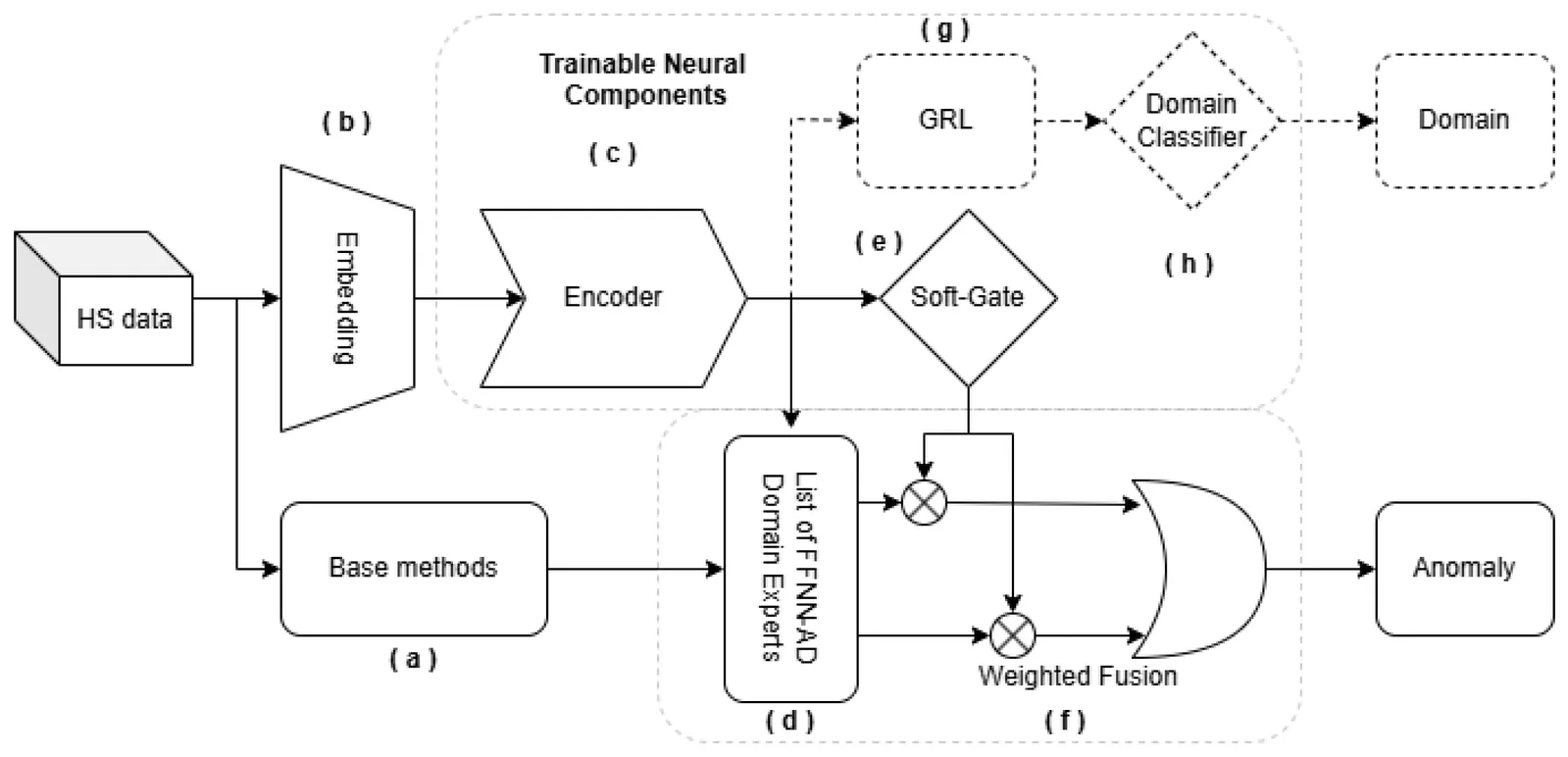
Overview of the proposed GMoE-AD framework. The dotted regions highlight trainable neural components, while (**a**,**b**) are fixed. The system consists of: (**a**) base methods that produce initial anomaly detection maps; (**b**) HyperSL embedding including a Universal Spectral Tokenizer and Spectral Wavelength Positional Encoding; (**c**) HyperSL encoder, which, together with (**b**), forms the unified feature-extraction backbone; (**d**) a group of FFNN-AD neural experts; (**e**) soft-gate network that predicts neural-expert weights from latent features; (**f**) weighted fusion module to produce the final anomaly prediction; (**g**) Gradient Reversal Layer (GRL); and (**h**) domain classifier, which, together with (**g**), enforces domain-invariant feature learning during training (Note that the dashed connections indicate training-only paths. The GRL (**g**) and domain classifier (**h**) are removed at inference).

To address these challenges, we propose **Generalized MoE-AD (GMoE-AD)**, a unified Mixture-of-Experts (MoE) framework for cross-domain HS anomaly detection, which extends ensemble-based detection to a cross-domain scenario. The proposed model leverages a pretrained HS foundation model [25] as a shared feature extraction backbone to learn transferable spectral–spatial representations across heterogeneous datasets. Instead of relying on dataset-specific models, the proposed framework, along with the backbone, employs a set of neural experts. In this work, each neural expert is a learnable component within the mixture architecture, and the router assigns input-dependent weights to their outputs. Figure 1 provides a technical overview of the framework, showing how base anomaly detectors, a shared hyperspectral foundation model, neural expert networks, soft-gated neural-expert fusion, and training-time domain-adversarial learning are integrated into a single pipeline. Unlike our previously developed Greedy Ensemble Anomaly Detector (GE-AD) [16], these neural stacking ensembles (FFNN-AD) employ a feedforward neural network (FFNN) as the meta-model and use extracted features from the backbone along with the detection output of a set of unsupervised HS-AD methods as inputs to the meta-model. These FFNN-AD neural experts are jointly trained with a MoE gating network [26] in an end-to-end manner using the training portions of all datasets. The MoE’s soft-gating network assigns input-dependent weights to the neural-expert outputs, enabling soft sample-adaptive routing without requiring explicit domain information at inference time.

To further enhance robustness, we incorporate domain adaptation [27,28] via a Gradient Reversal Layer (GRL) [29] during training to encourage domain-invariant feature learning. This allows the model to leverage shared structure across the training datasets while mitigating dataset-specific effects. Unlike previous ensemble methods that rely on models tailored to specific datasets, this framework is trained once in a unified manner and can perform inference without requiring knowledge of the domain or dataset identity. Generalization to a dataset excluded from training is evaluated separately using leave-one-dataset-out (LODO) evaluation. We also evaluate a drift-aware test-time adaptation (DTA) variant as an optional deployment mode, separately from the primary frozen model; its method and results are presented in Section 3.10 and Section 4.4, respectively.

In addition, we revisit HS unmixing-based anomaly detection and address a known limitation: its reliance on prior knowledge of target anomaly spectra. We propose an improved formulation that characterizes small, spectrally distinct objects as anomalies, thereby mitigating failures observed when target spectra are unknown or absent [30]. This improved unmixing-based anomaly detector is integrated as a base method of the FFNN-AD framework, enhancing detection robustness in unknown-target scenarios.

We evaluate the proposed framework on seven real-world HS datasets collected from four different sensors under all-domain training and leave-one-dataset-out settings. Under all-domain training, the model uses the training portions of all seven datasets and produces anomaly scores without access to domain identity or dataset-specific information at inference. In this setting, GMoE-AD achieves a macro-averaged F1-score (F1-macro) of 0.821, an average area under the receiver operating characteristic curve (ROC-AUC) of 0.943, and an area under the precision–recall curve (PR-AUC) of 0.623. These results measure performance on datasets represented during training; unseen-dataset performance is assessed by LODO. Single-domain models can transfer effectively among related datasets, but their performance can degrade under stronger sensor and scene shifts, as observed for Arizona. GMoE-AD maintains reliable performance without requiring prior knowledge of the target-domain identity, making it suitable for practical cross-domain deployment. These findings also provide new insights into the interaction between ensemble learning, transfer learning, and domain-adaptive architectures for robust HS-AD.

The rest of this paper is organized as follows. Section 2 reviews the background and related state-of-the-art techniques relevant to our work. Section 3 describes the proposed GMoE-AD architecture. In Section 4, we evaluate our method on seven real-world datasets to assess its accuracy and efficiency, and Section 5 discusses the results and observations. Finally, we summarize the findings of this paper and future directions in Section 6.

## 2. Related Work

This section summarizes the work most relevant to GMoE-AD. We first outline the challenge of cross-domain generalization in HS-AD and review domain-adversarial learning. We then discuss MoE architectures and recent HS-AD methods, including how MoE can mitigate domain variability by allowing multiple experts to specialize in different data distributions and enabling implicit domain-aware representations within a unified architecture. We finally review test-time adaptation for domain drift at inference time without requiring access to labeled target data. Building on these ideas, our proposed GMoE-AD framework uses MoE to capture heterogeneous domain characteristics during training while maintaining domain-agnostic frozen inference. An optional DTA variant is evaluated separately to examine whether test-time updates improve cross-domain generalization in HS anomaly detection.

### 2.1. Cross-Domain Generalization and Domain-Adversarial Learning

Domain adaptation and domain generalization methods aim to improve robustness when training and deployment distributions differ. A common strategy is to learn representations that remain discriminative for the target task while reducing sensitivity to domain-specific nuisance factors. Domain-adversarial neural networks implement this idea by coupling a task head with an auxiliary domain classifier through a Gradient Reversal Layer (GRL), so that the feature extractor is trained to support the task while making domain prediction difficult [29].

This idea is particularly relevant for cross-domain HS-AD because spectral responses can vary with sensor characteristics, illumination, atmospheric effects, acquisition geometry, and scene composition. If the feature extractor overfits to these dataset-specific factors, anomaly scores may degrade when deployed on a new scene or sensor.

Most existing HS-AD methods are still developed under a single-domain assumption, where training and testing are performed on the same dataset. Recent methods improve detection through enhanced background modeling, reconstruction, and spatial–spectral representation learning, including plug-and-play denoising convolutional neural network regularization, masked autoencoding, Transformer-based learning, and Mamba-based multiscale reconstruction [13,17,18,19,20,31]. Although these reconstruction and representation-learning methods provide strong performance within individual scenes or datasets, they are typically not designed for a domain-agnostic setting in which a single model is trained using heterogeneous datasets and deployed without dataset identity.

Several studies have explored robustness through improved background modeling and hybrid learning strategies. Collaborative representation and tensor-based approaches enhance resilience to complex spectral distributions [32,33], while Transformer-based reconstruction methods demonstrate robustness to noise and weak anomalies [19]. Transfer learning has also been introduced to address domain shift by leveraging labeled source-domain data and adapting to a target domain [21]. However, such approaches assume a predefined source–target setting and do not learn a unified representation across multiple heterogeneous domains.

Recent work such as the Spatially Informed Model (STIM) [34] emphasizes robustness through spatially informed statistical modeling and multi-feature scoring, achieving strong performance across diverse scenes in a fully unsupervised manner. Other recent methods have explored transfer-enhanced Transformer learning [22], continual cross-domain learning [23,24], and hyperspectral foundation models trained at scale [35]. These studies indicate a growing interest in transferable hyperspectral representations. Nevertheless, most recent HS-AD methods either focus on improving single-dataset accuracy, assume sequential task adaptation, or require a predefined source–target formulation. They therefore differ from the domain-agnostic deployment setting considered in this paper.

Our setting differs from conventional source–target domain adaptation. Our proposed GMoE-AD framework implicitly targets domain generalization. We evaluate in two complementary settings: (i) all-domain training, where a single model is trained using the training portions of all datasets and then evaluated without domain identity at inference, and (ii) LODO evaluation, where the model is trained without access to a held-out target domain dataset and tested directly on that unseen target domain under the same domain-agnostic inference premise. The domain classifier and GRL are used only as training-time regularizers to encourage domain-robust features. Experiments are conducted on seven real-world hyperspectral datasets comprising twenty-two images from four sensors, demonstrating robust, domain-agnostic anomaly detection.

### 2.2. Mixture-of-Experts Architectures

The MoE framework was originally introduced to decompose a complex problem into sub-problems handled by specialized models, with a gating network responsible for expert selection [26]. In this formulation, each expert is optimized using a separate loss, and the gate imposes hard assignment constraints to route each input to a single expert.

Sparsely Gated MoE (SG-MoE) further improved scalability by activating only the top-*k* experts per input, enabling large increases in model capacity with limited computational overhead [36]. A key contribution was the load-balancing loss, which prevents expert collapse in token-based routing scenarios common in natural language processing.

MoE architectures have since been widely adopted in language and sequence modeling tasks. Zoph et al. [37,38] demonstrated that soft gating with top-*k* expert collaboration improves robustness and generalization across diverse linguistic domains. More recently, Feng et al. [39] proposed an MoE framework for autonomous driving that mitigates performance degradation under ambiguous guidance by weighting multiple experts rather than relying on a single network.

Unlike prior work, which primarily focuses on token- or trajectory-level routing, our work applies MoE principles to HS-AD, where domain shifts arise from sensor and dataset heterogeneity rather than semantic ambiguity. This distinction motivates a lightweight routing strategy and loss design for soft, input-adaptive expert weighting, as discussed in Section 3.4.

### 2.3. Test-Time Adaptation for Domain Drift

Test-time adaptation (TTA) aims to improve model robustness under distribution shifts that occur at inference time, where the test data (target domain) differ from the training data (source domain) [40]. TTA updates selected model components using unlabeled target samples during deployment.

A common approach in TTA relies on normalization-based adaptation. Batch Normalization (BN) [41] normalizes features using batch statistics during training while maintaining running estimates of population-level statistics for inference. BN has been widely used as an adaptation handle by updating either its running statistics or affine parameters at test time (target statistics). Test-time entropy minimization (TENT) adapts normalization affine parameters by minimizing prediction entropy, while continual test-time adaptation methods introduce stabilization mechanisms to reduce drift over long test streams [42,43]. Dynamic unsupervised adaptation (DUA) [44] instead updates BN statistics to align test-time feature distributions with those observed during training.

Normalization-based adaptation methods are effective for convolutional architectures using BN. However, Transformer-based models primarily utilize Layer Normalization (LN) [45], which normalizes features per sample and does not retain population statistics, limiting the direct use of BN-based TTA methods. Yet, entropy-minimization techniques like TENT can adapt LN’s affine parameters despite the lack of running statistics. Continual test-time adaptation (CoTTA) [43] demonstrates the effectiveness of this approach by updating LN parameters and incorporating temporal ensembling for stability. These findings indicate that normalization-based adaptation is still viable for LN-based models, though they do not clearly differentiate between population-level and representation-level shifts.

HS imagery makes uncontrolled test-time adaptation especially challenging because anomaly pixels are rare, spectral bands are highly correlated, and batch statistics can vary strongly across sensors and scenes. This can result in unstable parameter shifts and degraded anomaly contrast, particularly when distribution shifts are mild. While these methods help when the target distribution changes post-training, they risk negative transfer when adaptation is unnecessary or when the test batch primarily consists of background samples, as seen in HS-AD [46]. This highlights the need for selective or constrained adaptation strategies.

Recent TTA approaches enhance robustness by limiting adaptation to certain model components or adding regularization. Source hypothesis transfer (SHOT) [47] adapts only the classifier, freezing the feature extractor, while CoTTA [43] uses temporal ensembling and stochastic restoration for stable adaptation. However, these methods do not explicitly determine whether adaptation is needed for specific test samples or distributions.

Therefore, in this work, we report the frozen GMoE-AD model as the primary configuration and evaluate the proposed DTA method as an optional adaptive variant. The DTA module adjusts normalization-related parameters according to measured drift criteria, as described in Section 3.9 and Section 3.10 and evaluated in Section 4.4.

## 3. Materials and Methods

### 3.1. System Overview

Classical ensemble-based HS-AD methods assign fixed importance weights to base detectors learned from a single dataset. Because these weights implicitly encode dataset-specific statistical structure, performance degrades when the underlying spectral distribution shifts across sensors or scenes. In this work, sensor characteristics, atmospheric conditions, illumination, acquisition geometry, and scene composition are not modeled as independent physical variables; instead, they are treated as interacting factors that jointly shape the dataset-level domain distributions used for training and evaluation.

To address this limitation, we design a unified MoE framework for HS-AD, which extends ensemble-based detection to a cross-domain setting and combines adaptive neural expert routing with domain-robust representation learning. Figure 1 illustrates the overall architecture of the proposed generalized anomaly detector. The system follows a left-to-right processing pipeline consisting of base anomaly detectors, a unified feature-extraction backbone, stacking ensembles, and an MoE soft-gate router.

**Base Methods (a)** Each input HS image is processed by multiple base HS-AD methods to produce anomaly response maps. The outputs are concatenated into a feature vector b, which serves as input to the FFNN-AD neural experts. We used the following statistical HS-AD methods: Attribute and Edge-Preserving Filters (AED) [48], Fuzzy Cluster-Based Anomaly Detection (FCBAD) [49], HS-AD With Kernel iForest (KIFD) [50], Kernel-RX Algorithm (KRX) [51], and Local Summation Unsupervised Nearest Regularized Subspace with an Outlier Removal Anomaly Detector (LSUNRSORAD) [52]. We also used one hyperspectral unmixing-based AD, and this method is described in Section 3.2.
**Feature Embedding and Encoder (Backbone, b–c)** In parallel, the HS input is passed through the pretrained foundation model HyperSL [25]. HyperSL is selected due to its spectral-aware architecture and strong cross-dataset generalization capability, which are desirable for domain-robust anomaly detection (see Section 3.3 for further discussion). The HyperSL embedding stage (b), composed of a Universal Spectral Tokenizer and Spectral Wavelength Positional Encoding, is kept frozen, while the HyperSL encoder (c) is fine-tuned during training. Together, they form the shared backbone and collectively produce the latent feature representation $f_{l}$. These latent features are consumed by our FFNN-AD neural experts. They are also used for neural-expert routing (Section 3.4) and domain-invariant feature learning (Section 3.6).
**FFNN-AD Neural Experts (d)** A set of FFNN-AD neural experts is employed, each implemented as a lightweight two-layer feedforward neural network that takes both the base-method feature vector b and the latent feature $f_{l}$ as input. Here and throughout the paper, a neural expert denotes a trainable subnetwork within the MoE architecture, not a base detector. These neural experts are soft-specialized, allowing multiple neural experts to collaboratively address anomaly detection across domains. All neural experts are trained jointly in an end-to-end manner with the rest of the framework (Section 3.4.1).
**Soft-Gate Network (e)** A lightweight gating network takes $f_{l}$ and a three-statistic spectral fingerprint derived from the input HS pixel and produces a probability distribution over the neural experts (Section 3.4.2). These probabilities indicate the relevance of each neural expert for the input and are used as fusion weights.
**Weighted Fusion (f)** The final anomaly score is computed as a weighted sum of the top-*k* neural-expert outputs (Section 3.4.3). This allows multiple high-confidence neural experts to contribute proportionally to the final prediction.
**Domain-Adversarial Path (g–h)** During training, the latent feature $f_{l}$ is passed through (g) a GRL and (h) a domain classifier. This branch predicts dataset-domain labels while reversing gradients during backpropagation, encouraging the backbone to learn domain-invariant representations (Section 3.6). During inference, this branch is removed; the model does not accept domain labels as input and only outputs the anomaly score.

### 3.2. Base Method—Unmixing

Endmembers are unique spectra representing the materials in an HS image. However, the spectral signature of a pixel can contain a linear combination of multiple endmembers [53]. Spectral unmixing algorithms such as Harsanyi–Farrand–Chang (HFC) [54], N-FINDR [55], and Fully constrained least-squares (FCLS) [56] decompose the measured spectra into a collection of endmembers, determine the proportion of each endmember in the pixel, and produce the *abundance map* using the identified endmembers. Younis et al. [30] used spectral information divergence (SID) [57] to measure spectral similarity between pixels and reference spectra, analogous to HS target detection. We can obtain these reference spectra from datasets such as the ECOSTRESS spectral library [58,59] or extract them from annotated HS images within the same dataset or from other datasets. However, this method is unusable when target spectra are unknown. Thus, we propose a method that yields a set of anomalous abundances representing fewer than a small percentage (*p*) of pixels in a scene, since anomalies are typically rare, and use it as one of the input base methods. We found that p<20% provides a stable trade-off between sparsity and coverage across datasets [60]. Unlike SID-based methods that require known target spectra, our formulation operates without prior target knowledge and derives anomalous references directly from the scene. Algorithm 1 summarizes the proposed target-free anomalous abundance generation procedure, which replaces predefined target spectra with a rarity-based selection of abundance responses.
**Algorithm 1** Anomalous abundance generation via spectral unmixing.**Require:** HS image cube $X\in R^{H\times W\times B}$, wavelengths λ, unmixing method (FCLS), rarity threshold p=20%**Ensure:** Anomalous abundance map $A_{\mathrm{anom}}\in R^{H\times W}$1:Ensure/Construct HS cube X using spectral bands λ2:Estimate number of endmembers *K* using HFC with noise whitening3:Extract endmembers $E\in R^{K\times B}$ via N-FINDR4:**for all** pixels $x_{u,v}\in X$ **do**5:    Estimate abundance vector $a_{u,v}\in R^{K}$ using FCLS6:**end for**7:Assign dominant endmember class map $c_{u,v}=\arg\max_{k}a_{u,v,k}$8:Compute pixel percentage for each endmember class9:Select rare classes $C_{\mathrm{anom}}=\left\{k|\mathrm{percentage}\left(k\right)<p\right\}$10:Sort $C_{\mathrm{anom}}$ in ascending order of percentage11:Initialize $A_{\mathrm{anom}}\leftarrow 0$12:Initialize weighting factor ρ←1.013:**for all** endmember indices $k\in C_{\mathrm{anom}}$ **do**14:    **for all** pixels (u,v) such that $c_{u,v}=k$ **do**15:        $A_{\mathrm{anom}}\left(u,v\right)\leftarrow \rho \cdot a_{u,v,k}$16:    **end for**17:    ρ←ρ−0.0518:**end for**19:**return** $A_{\mathrm{anom}}$

In Algorithm 1, *H*, *W*, and *B* denote the image height, image width, and number of spectral bands, respectively, while $a_{u,v,k}$ denotes the abundance of endmember *k* at spatial location (pixel) (u,v). After initializing the output to zero, the procedure visits the selected endmember classes from rarest to less rare, writes the corresponding weighted FCLS abundances to their original pixel locations, and reduces the weight by 0.05 for each successive class; pixels outside the selected rare classes remain zero. Thus, $A_{\mathrm{anom}}\left(u,v\right)$ retains the original two-dimensional spatial arrangement of the HS image, and rarer endmember classes are emphasized by assigning larger weights, ensuring that the most infrequent spectral responses contribute more strongly to the anomalous abundance map. For example, in a 100×100 scene, abundance vectors are first estimated for all pixels; endmember classes representing fewer than 20% of pixels (2000) are then retained, yielding sparse abundance maps that highlight small, spectrally distinct objects. The resulting anomalous abundance map then serves as a base anomaly detector and is combined with other base methods within the proposed ensemble framework.

### 3.3. Foundation Model as Backbone

We use an HS Vision Transformer (ViT)-based foundation model called HyperSL [25] as our feature-extraction backbone because of its unified spectral representation. This allows transfer across different datasets and sensors with minimal changes to the architecture. HyperSL was pretrained on about 200 million spaceborne HS samples from two sensors, EnMAP (the Environmental Mapping and Analysis Program) and DESIS (the DLR Earth Sensing Imaging Spectrometer). This pretraining transfers knowledge from related source domains to improve generalization in target domains with limited labeled data.

The backbone comprises a Universal Spectral Tokenizer, Spectral Wavelength Positional Encoding, and a three-block Transformer encoder. The tokenizer maps diverse spectral vectors into a shared token space, while the wavelength-aware positional encoding aligns features across spectral ranges. As illustrated in Figure 1, these components form the embedding stage (component b) and the encoder (component c), which produce latent features. The resulting representations are provided to the soft gate module for routing and to the GRL and domain classifier for domain-adversarial learning.

During training, the HyperSL encoder is fine-tuned jointly with the downstream modules, while the tokenizer and positional encoding components remain fixed.

### 3.4. Mixture-of-Experts Framework

We propose a unified MoE framework for HS-AD, which extends ensemble-based detection to a cross-domain scenario. Instead of relying on dataset-specific models, the proposed framework learns a set of neural expert networks that are combined through input-adaptive routing after joint training on the training portions of all datasets. Given an input HS sample, a soft-gate network adaptively selects and combines a subset of neural experts to produce the final anomaly score. Domain labels are used only in training-time auxiliary objectives for feature alignment and routing-diversity regularization; they are not used to assign neural experts to specific domains and are not required at inference time. As a result, neural-expert selection is performed solely via the gating network based on the input features, enabling domain-agnostic inference when the test distribution differs from the training domains.

#### 3.4.1. Neural Experts

Let $\left\{E_{1},E_{2},\ldots ,E_{N_{e}}\right\}$ denote a pool of $N_{e}$ FFNN-based AD neural experts, as shown in component d of Figure 1. Each neural expert produces an anomaly prediction as defined in Equation (1):

(1) $$a_{i}\left(x\right)=E_{i}\left(b\left(x\right),f_{l}\left(x\right)\right),\;i=1,\ldots ,N_{e},$$

where b(x) is the base-method feature vector and $f_{l}\left(x\right)$ is the latent feature representation from the backbone for an input HS pixel x.

**Figure 2:**
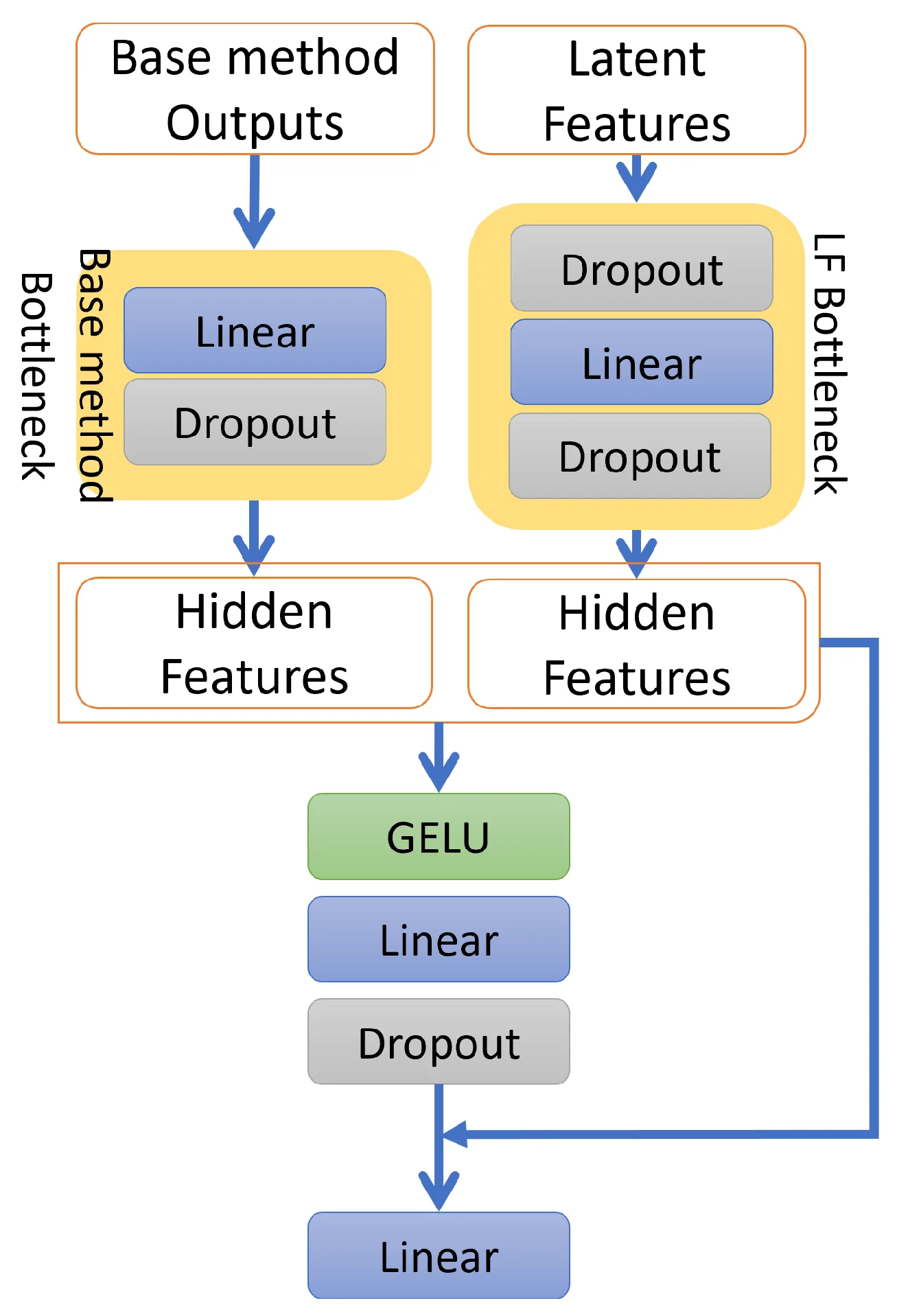
FFNN-AD neural expert.

Figure 2 shows the outline of our proposed FFNN-AD neural expert. All neural experts share the same architecture but are initialized independently with different random parameter initializations. No domain-specific pretraining or manual neural-expert-to-domain assignment is performed. During training, all FFNN-AD neural experts are jointly optimized using samples from all datasets (domains).

The MoE framework [26] learns a soft gating function that assigns input-dependent weights to the neural experts. Domain specialization is encouraged but not imposed: the load-balancing loss promotes broader neural-expert utilization, while the domain-diversity loss encourages routing distributions to differ across heterogeneous training domains. Consequently, the neural experts can learn complementary responses to background distributions and anomaly characteristics, but they should be interpreted as soft functional components rather than one-to-one domain-specific detectors. These domain characteristics may reflect the combined effects of sensor response, atmospheric variability, illumination, and scene composition, but the present model does not attempt to disentangle those factors individually. The gating mechanism then combines the most relevant neural experts for each input.

#### 3.4.2. Soft-Gate Network

**Figure 3:**
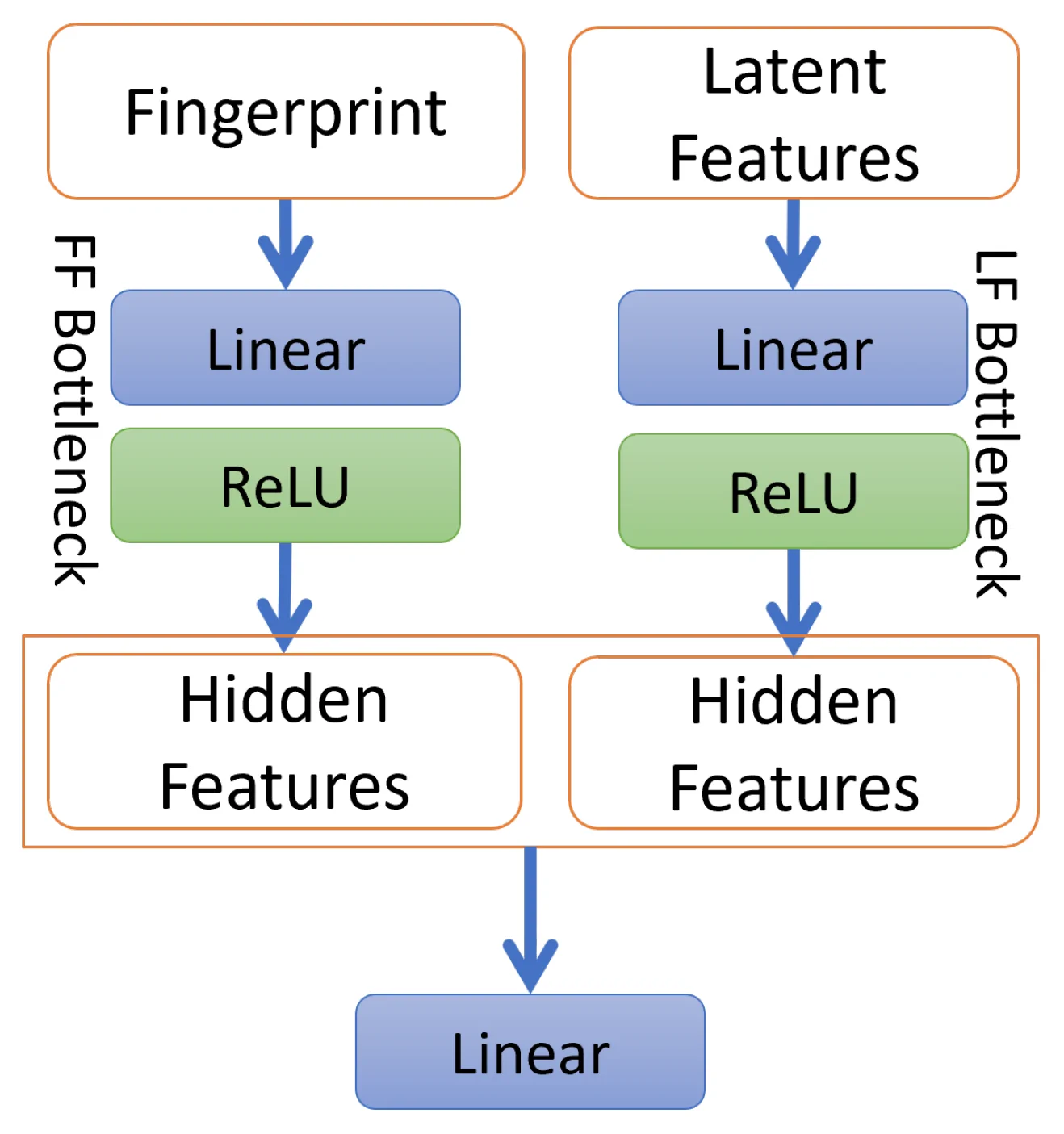
Soft-gate network combining latent features with the three-statistic spectral fingerprint.

Figure 3 shows the outline of our proposed lightweight feedforward soft-gate network G(·). It takes the latent feature $f_{l}$ along with simple spectral statistics—mean, standard deviation, and maximum—calculated from the input HS pixel. These statistics form the lightweight spectral fingerprint defined in Equation (2):

(2) $$s\left(x\right)={\left[\mathrm{mean}\left(x\right),\mathrm{std}\left(x\right),\max\left(x\right)\right]}^{T}$$

This fingerprint complements the learned latent representation and offers a compact summary of the spectral distribution, providing additional low-level cues about the data source and its domain that might not be fully captured by the learned latent representation, especially during cross-domain spectral shifts. As illustrated in Figure 3, separate linear/ReLU branches project $f_{l}$ and s(x), and their concatenated representation is mapped to raw gate logits z. The logits are temperature-scaled and normalized to obtain non-negative expert weights (see Figure 1, component e):

(3) $$z=G\left(f_{l},s\left(x\right)\right),\;w_{i}=\frac{\exp(z_{i}/T)}{\sum_{j=1}^{N_{e}}\exp\left(z_{j}/T\right)},\;\sum_{i=1}^{N_{e}}w_{i}=1,$$

In Equation (3), *T* is the routing temperature. The resulting weights represent the relevance of each expert for the given input and are subsequently used for top-*k* selection.

#### 3.4.3. Top-k Neural-Expert Selection

To reduce interference from weakly related domains, only the top-*k* gating weights are retained [37,38], as defined in Equation (4):

(4) K=TopK(w,k).

Experts outside K are ignored for a given input to reduce contributions from unrelated experts. Zoph et al. [38] recommend k=2, and we observe that this choice provides stable performance across heterogeneous datasets while helping to avoid routing collapse (i.e., dominance by a single expert). Although prior work reports small gains from top-(n+1) over top-*n* routing, we found this improvement to be negligible in our setting. Using only the top-1 expert reduces the model to hard routing, increasing sensitivity to uncertain routing decisions and amplifying routing errors. Conversely, activating all experts introduces contributions from weakly related domains, leading to over-smoothing of anomaly scores and reduced discriminative contrast.

### 3.5. Weighted Prediction Fusion

The final anomaly prediction is computed as a weighted sum of the selected experts’ outputs (see Figure 1, component f). Given the top-*k* selected experts K with gating scores $w_{i}$, the weights are first re-normalized over the selected set according to Equation (5):

(5) $${\tilde{w}}_{i}=\frac{w_{i}}{\sum_{j\in K}w_{j}}\;,\;\mathrm{for}\;i\in K.$$

The final anomaly score is then obtained from Equation (6):

(6) $$a_{\mathrm{final}}\left(x\right)=\sum_{i\in K}{\tilde{w}}_{i}\;a_{i}\left(x\right).$$

This re-normalization ensures that the selected high-confidence experts contribute proportionally while preserving a convex combination ($\sum_{i\in K}{\tilde{w}}_{i}=1$), producing a single, unambiguous anomaly score that is robust to cross-sensor and cross-dataset variation.

Although inspired by sparse and soft-gated MoE architectures [36,37], our approach uses FFNN-AD subnetworks as lightweight neural experts. Routing occurs at the sample level rather than the token or trajectory level, and expert choice is inferred from input features rather than from explicit domain labels at inference time.

### 3.6. Training-Time Domain Adaptation and Generalization

We formulate the proposed framework as a multi-task learning problem that jointly optimizes the objectives of anomaly detection and domain classification. Domain adaptation is implemented using the GRL [29], which is placed between the backbone and the domain classifier. During training, the domain classifier is trained to correctly identify the dataset for each sample, while GRL inverts the gradients before they reach the backbone, thereby encouraging the backbone to learn feature representations that remain discriminative and informative for anomaly detection but insensitive to dataset-specific differences. The objective is therefore to reduce dataset-specific information in the shared features. In addition, we investigate domain generalization by training on multiple source domains to capture invariant factors shared across datasets [61].

### 3.7. Training and Inference

Training is performed end-to-end, reflecting the unified design of the proposed architecture. Unlike the original GE-AD formulation, which employs frozen experts [16], we replace the meta-model with a trainable feedforward network, enabling joint optimization; each FFNN-AD expert is trained jointly with the rest of the framework on labeled anomaly data from all datasets and is updated through the MoE mechanism. This end-to-end training allows experts to adapt dynamically during learning; its unseen-dataset behavior is measured by LODO.

The HyperSL backbone is initialized from a pretrained foundation model [25]. During training, the backbone is first frozen for 2–3 warm-up epochs to stabilize the gating and downstream modules, after which it is fine-tuned jointly with the gating network and the domain classifier. Due to inconsistent spectral dimensions across data sources, it is not possible to load them into the same batch for training the output model. To mitigate class imbalance and domain-induced distribution bias, we employed three complementary data sampling strategies (minority-class, domain-aware, and spectral-dimension) in the data loading pipeline. Spectral-dimension sampling improves robustness to varying spectral dimensionality (57–300 channels) by exposing the model to inputs with different channel counts. During training, each mini-batch is constructed with a fixed spectral dimensionality (i.e., all samples in a batch share the same number of channels), while the dimensionality varies across batches. Given an input HS sample, the backbone extracts latent features that the gating network uses to assign weights to experts and that the domain classifier uses for domain-adversarial learning. During this stage, the model is optimized using a weighted combination of classification, domain, and routing-related losses, described below. The GRL ensures that the backbone learns representations that are discriminative for anomaly detection while remaining invariant to dataset-specific characteristics.

Training minimizes the overall loss objective, while macro-F1 at a threshold of 0.50 is monitored throughout optimization as an evaluation metric. We use the AdamW optimizer [62] with an initial learning rate of $5\times 10^{-4}$ and a cosine annealing schedule with warm restarts [63], configured with $T_{0}=5$, $T_{mult}=2$, and $\eta_{\min}=1\times 10^{-8}$. All components are trained using mixed precision (bfloat16) [64,65] to reduce memory and computational cost while preserving numerical stability. The neural implementation used Python 3.12 and PyTorch 2.7.1 with CUDA 12.6. Training was conducted on one NVIDIA H100 80 GB graphics processing unit (GPU; NVIDIA Corporation, Santa Clara, CA, USA).

Unless otherwise specified, the reference implementation uses fused HyperSL and six-base-detector inputs, four FFNN neural experts, learned top-2 routing, and a routing temperature of T=1.5. Models are trained for up to 50 epochs, with early stopping. For the primary five-run evaluation, the training launcher selected a batch size of 5096 from the available GPU memory of 81,559 mebibytes (MiB). The controlled architecture and routing-loss experiments instead used a fixed batch size of 1024 and three fixed random seeds: 8264, 6603, and 1954. DTA is disabled in these experiments and is evaluated separately in Section 4.4.

During inference, the F1-macro decision threshold is selected on the validation set. Only the backbone, gating network, and FFNN-AD neural experts are active; the domain classifier and GRL are disabled, resulting in a single anomaly score per input sample.

### 3.8. Loss Function

Our model aims to achieve several objectives simultaneously. The overall loss combines anomaly classification, domain-adversarial, and regularization terms, with components activated or emphasized at different training stages. The anomaly classification loss helps accurately distinguish normal and anomalous samples. The domain-adversarial loss is another key loss; it helps the learned features generalize across domains rather than relying on domain-specific artifacts. As the model uses multiple neural experts, additional regularization terms guide the routing mechanism to prevent routing collapse and expert regularization to stabilize expert-specific outputs.

**Domain-Weighted Anomaly Classification** The primary supervision is a domain-weighted binary cross-entropy loss with logits applied at the sample level:
  (7) $$L_{cls}=\frac{1}{N}\sum_{i=1}^{N}\omega_{d(i)}\;\ell_{BCE}\left(y_{i},{\hat{y}}_{i}\right)$$
  where *N* is the total number of training samples, d(i)∈D denotes the domain label of sample *i*, D is the set of training domains, and $\omega_{d(i)}$ is a domain-specific weight applied to each sample.
  The loss $\ell_{BCE}$ is a class-weighted binary cross-entropy loss defined as
  (8) $$\ell_{BCE}\left(y,\hat{y}\right)=-\left[\alpha \;y\log\sigma \left(\hat{y}\right)+\left(1-y\right)\log\left(1-\sigma \left(\hat{y}\right)\right)\right]$$
  where σ(·) is the sigmoid function and α is a positive class weight that up-weights anomaly samples. In practice, α is applied only to anomaly samples (i.e., y=1), consistent with the pos_weight formulation to address class imbalance.
  The domain weight $\omega_{d}$ is defined as an inverse-frequency scaling factor based on the number of samples $n_{d}$ in domain *d*, i.e., $\omega_{d}\propto \frac{1}{n_{d}}$, followed by normalization and rescaling to a bounded range with clipping to ensure stability.
  This formulation decouples domain imbalance (handled by $\omega_{d}$) from class imbalance (handled by α), where $\omega_{d}$ scales all samples from a domain and α affects only anomaly samples. The contribution of $L_{cls}$ is gradually increased during training to stabilize optimization in early stages.
**Domain-Adversarial Loss** A multi-class cross-entropy (CE) domain-classification loss ($L_{grl}$) is applied to the domain-prediction head after a warm-up period using GRL. The loss is annealed as
  (9) $$L_{grl}=\frac{1}{t+1}\cdot \ell_{CE}\left(d_{i},{\hat{d}}_{i}\right)$$
  where *t* is the training epoch. The domain-adversarial loss, applied via the GRL, encourages domain-invariant feature learning.
**Load Balancing Loss** To prevent expert collapse during early training, we apply a load balancing loss ($L_{lb}$):
  (10) $$L_{lb}=N_{e}\sum_{j=1}^{N_{e}}p_{j}f_{j}$$
  where $N_{e}$ is the number of neural experts, $p_{j}$ is the average routing probability, and $f_{j}$ is the fraction of samples routed to neural expert *j*. The factor $N_{e}$ normalizes the loss such that its minimum value is 1 under uniform expert utilization ($p_{j}=f_{j}=1/N_{e}$), making the scale independent of the number of neural experts. Higher values indicate imbalance, where a subset of neural experts dominates. This term is active only during the initial training phase. Expert collapse refers to the scenario where most samples are routed to a small subset of neural experts [64].
**Domain Diversity Loss** To encourage routing diversity across heterogeneous training domains without assigning experts manually, we introduce a margin-based diversity loss with masked distance computation:
  (11) $$L_{div}=\frac{1}{\left|P\right|}\sum_{(d,d^{\prime })\in P}\max\left(0,\;m-{∥\left({\tilde{p}}_{d}-{\tilde{p}}_{d^{\prime }}\right)⊙M_{d,d^{\prime }}∥}^{2}\right)$$
  where $P=\{\left(d,d^{\prime }\right)|d,d^{\prime }\in D,\;d\neq d^{\prime }\}$ denotes the set of distinct domain pairs, including both comparisons among current batch-wise domain distributions and comparisons between batch distributions and their corresponding historical domain means. |P| is the number of such pairs. ${\tilde{p}}_{d}$ and ${\tilde{p}}_{d^{\prime }}$ are normalized routing distributions, $M_{d,d^{\prime }}$ is a top-*k* mask selecting dominant experts, and *m* is an adaptive margin.
  This loss consists of two components: (i) separation between current batch-wise domain distributions, and (ii) separation between batch distributions and historical domain means. The historical means are estimated via an exponential moving average (EMA) and normalized to form valid distributions. Domain weights are applied when comparing against historical means.
**Entropy Regularization** An entropy regularization term is applied to the router:
  (12) $$L_{H}=-E\left[\sum_{j}p_{j}\log p_{j}\right]$$
  This term is subtracted from the loss to encourage confident routing and is only applied during early training.
**Expert Regularization** When expert-specific outputs are enabled, an $\ell_{2}$ regularization term is applied to expert logits to stabilize training:
  (13) $$L_{expr}=E\left[∥z_{\mathrm{expr}}{∥}^{2}\right]$$
**Final Objective** The total loss is
  (14) $$L=\lambda_{cls}\left(t\right)L_{cls}+\lambda_{dom}L_{grl}+\lambda_{lb}L_{lb}+\lambda_{div}L_{div}-\lambda_{H}L_{H}+\lambda_{expr}L_{expr}$$
  The fixed loss weights are $\lambda_{dom}=0.02$, $\lambda_{lb}=0.1$, $\lambda_{div}=1.0$, $\lambda_{H}=0.1$, and $\lambda_{expr}=0.001$. These values are held constant across the controlled three-seed experiments; the routing-loss ablation in Section 4.7 removes the corresponding terms individually and jointly rather than performing an exhaustive loss-weight search.
  Intuitively, the different loss terms serve complementary purposes. The classification loss $L_{cls}$ drives the primary task of anomaly detection. The domain-adversarial loss $L_{grl}$ encourages features that generalize across domains. The routing-related losses ($L_{lb}$, $L_{div}$, and $L_{H}$) regulate how samples are assigned to experts by preventing expert collapse, encouraging routing diversity, and encouraging confident routing decisions. The expert regularization term $L_{expr}$ stabilizes expert-specific outputs. The weighting coefficients balance these objectives throughout training.
  Each component is activated conditionally. Load balancing and entropy regularization are applied during early training, while domain diversity and adversarial learning are introduced after a warm-up period. The classification weight $\lambda_{cls}\left(t\right)$ increases over time, enabling stable optimization followed by stronger task supervision.

### 3.9. Test-Time Inference

After training, GMoE-AD can be deployed in a frozen domain-agnostic inference mode. In the primary configuration, all model parameters are fixed. Continuous anomaly-score inference does not require target-domain labels, dataset identity, or dataset-specific calibration. The validation-based calibration used for threshold-dependent metrics is described in Section 3.12.

For each test sample, the HyperSL backbone extracts the latent feature representation, the soft-gate network predicts neural-expert weights from the input features, the top-*k* neural experts are selected, and the final anomaly score is computed by weighted fusion. The GRL and domain classifier are training-only components and are removed during inference. Therefore, the deployed frozen model consists only of the backbone, router, FFNN-AD neural experts, and weighted fusion module.

We also evaluate a drift-aware test-time adaptation (DTA) variant as an optional deployment mode, as described in Section 3.10. DTA is applied only to unlabeled test samples and is designed to adapt conservatively when the incoming batch exhibits measurable distribution drift. The module monitors deviations in normalization statistics and representation-level responses, and updates only selected normalization statistics and affine parameters based on the drift level, while keeping the main anomaly-detection architecture fixed. This constrained design avoids full-network test-time retraining and reduces the risk of unstable adaptation under severe anomaly-background imbalance. The frozen model is used as the main reported configuration, while the DTA variant is analyzed separately in Section 4.4.

### 3.10. Drift-Aware Test-Time Adaptation

The DTA variant uses a hierarchical adaptation strategy designed for HS-AD. Its goal is to adapt the model to distributional shifts that may occur at inference time while avoiding unnecessary or harmful updates when the test distribution closely matches the training distribution.

Given incoming test samples, we first estimate distributional drift relative to source-domain statistics computed during training. This drift estimation determines whether test-time adaptation should be enabled and, if so, which components of the model should be adapted. By conditioning adaptation on explicit drift measurements, the strategy is intended to limit over-adaptation and preserve anomaly-discriminative representations under stationary conditions.

The DTA framework operates hierarchically: when the detected drift is small, no adaptation is performed and source-domain statistics are preserved; as the magnitude of drift increases, progressively stronger adaptation mechanisms are selectively activated [42,44]. This design enables stable adaptation in high-dimensional HS settings, where indiscriminate normalization updates may otherwise degrade performance [46].

#### 3.10.1. Batch Normalization Drift Estimation

To quantify distributional shifts at the population level, we measure deviations between target-domain Batch Normalization (BN) statistics and the frozen source-domain BN statistics obtained after training. This approach is motivated by the moment-matching interpretation of BN [41] and its use in normalization-based test-time adaptation, but does not rely on entropy minimization to infer the presence or direction of shift.

Let a BN layer maintain frozen channel-wise source statistics $\left(\mu_{s}^{c},{\left(\sigma_{s}^{c}\right)}^{2}\right)$. Given a target batch $x_{t}\in R^{N\times C\times H\times W}$, we compute channel-wise target statistics:

(15) $$\mu_{t}^{c}=E_{n,h,w}\left[x_{t}^{n,c,h,w}\right],\;{\left(\sigma_{t}^{c}\right)}^{2}={\mathrm{Var}}_{n,h,w}\left[x_{t}^{n,c,h,w}\right]$$

We define the Batch Normalization drift score $D_{\mathrm{BN}}$ as a normalized channel-wise deviation:

(16) $$D_{\mathrm{BN}}=\frac{1}{C}\sum_{c=1}^{C}\left(\frac{|\mu_{t}^{c}-\mu_{s}^{c}|}{\sqrt{{\left(\sigma_{s}^{c}\right)}^{2}+\epsilon }}+\frac{|\sigma_{t}^{c}-\sigma_{s}^{c}|}{\sigma_{s}^{c}+\epsilon }\right)$$

where ϵ is a small constant for numerical stability. This score provides a scale-invariant, layer-wise indicator of covariate shift without requiring access to stored source data, unlike discrepancy-based measures such as maximum mean discrepancy (MMD) [66].

#### 3.10.2. Layer Normalization Drift Estimation

While BN captures population-level distributional shifts, Transformer-based architectures rely primarily on Layer Normalization (LN), which operates at the sample level and is invariant to batch statistics. As a result, BN-based drift measures alone are insufficient to detect representation-level shifts affecting LN-based models.

To estimate such representation drift, we use the energy score [67], computed from the anomaly classifier logits as defined in Equation (17):

(17) $$E_{t}=-\log\sum_{k=1}^{K}\exp\left(z_{k}\right)$$

where $z_{k}$ denotes the logit corresponding to class *k* produced by the anomaly classifier head. Let $E_{s}$ and $E_{t}$ denote the energy scores computed on the source (training/validation) domain and the target domain, respectively. By storing the mean energy score on a validation set during training, denoted as $E[E_{s}]$, we define the Layer Normalization drift score $D_{\mathrm{LN}}$ as

(18) $$D_{\mathrm{LN}}=\left|E\left[E_{t}\right]-E\left[E_{s}\right]\right|$$

This metric captures shifts in the model’s confidence distribution and provides a lightweight indicator of representation-level drift relevant to LN-based architectures.

#### 3.10.3. Drift-Aware Adaptation Policy

Using the BN- and LN-based drift scores, we define a drift-aware adaptation policy that selectively activates test-time adaptation mechanisms. Given predefined thresholds $\tau_{1}$, $\tau_{2}$, and $\tau_{3}$, the policy operates as follows:

**(a) No domain shift ($D_{\mathrm{BN}}<\tau_{1}$):** The target distribution closely matches the source distribution. All normalization statistics are restored to their source values, and no adaptation is performed.
**(b) Mild shift ($\tau_{1}\leq D_{\mathrm{BN}}<\tau_{2}$):** Adaptation is restricted to updating BN running statistics, correcting low-order distributional mismatches while preserving learned representations [44].
**(c) Severe shift ($D_{\mathrm{BN}}\geq \tau_{2}$):** Stronger adaptation is enabled by updating BN affine parameters to improve prediction confidence [42].
**(d) Representation drift ($D_{\mathrm{LN}}\geq \tau_{3}$):** When representation-level drift is detected, normalization affine parameters (including Layer Normalization parameters in Transformer-based components) are updated to reduce predictive uncertainty. If both severe BN drift and LN drift are present, adaptation is applied jointly while avoiding redundant updates.

For the reported DTA evaluation, the fixed thresholds are $\tau_{1}=0.05$, $\tau_{2}=0.10$, and $\tau_{3}=0.10$. Because the DTA calibration metadata was not explicitly saved during training, the source energy reference is computed from 4096 source-validation samples. The source BN references are captured from the running statistics restored with each frozen checkpoint and are not estimated from those 4096 samples. Test-time updates use minibatches of 1024 samples with an affine-parameter learning rate of $10^{-4}$. Depending on the measured drift, BN running statistics and selected BN/LN affine parameters are adapted, with at most one gradient-based entropy-minimization update per adaptation minibatch. These values are fixed settings evaluated against frozen inference; an exhaustive sensitivity study of the DTA thresholds is not performed.

Overall, the DTA policy conditions normalization updates on the measured drift. The reported experiments evaluate its effect on both convolutional and Transformer-based components using the fixed settings above.

### 3.11. Datasets

We evaluated the effectiveness of the anomaly detection algorithms using seven datasets drawn from five benchmark collections: the Airport–Beach–Urban (ABU) dataset [48,68], the San-Diego dataset [69,70], the Salinas dataset [71], the HYDICE-Urban dataset [72], and the Arizona dataset [73].

The ABU dataset contains three subsets: Airport, Beach, and Urban. Following the preprocessing pipeline described by Kang et al. [48], noisy bands were identified using a noise-estimation method [74], additional preprocessing steps were applied, and unusable bands were discarded across all subsets. The ABU-Airport subset includes four airport scenes of size 100×100 pixels, extracted from larger Airborne Visible/Infrared Imaging Spectrometer (AVIRIS; Jet Propulsion Laboratory, Pasadena, CA, USA) images [75]. The AVIRIS sensor captures spectral radiance across 224 spectral bands covering wavelengths from 370 to 2510 nm. Three scenes have a spatial resolution of 7.1 m per pixel, while one scene has a resolution of 3.4 m per pixel. In this subset, anomalies correspond to airplanes. The ABU-Beach subset consists of four 100×100 and 150×150 pixel beach scenes acquired using AVIRIS and the Reflective Optics System Imaging Spectrometer (ROSIS-03), which was jointly developed by EADS (Ottobrunn, Germany), the German Aerospace Center (DLR; Oberpfaffenhofen, Germany), and the GKSS Research Centre (Geesthacht, Germany). The scenes have varying spatial resolutions. Anomalies include ships and other foreign objects in the sea. The ABU-Urban subset comprises five 100×100 pixel urban scenes collected with the AVIRIS sensor, each at a different spatial resolution. Anomalies in these images are primarily man-made structures located in semi-developed urban areas.

The San-Diego dataset, collected using AVIRIS, contains two 100×100 pixel images with a spatial resolution of 3.5 m per pixel. After removing bands with a low signal-to-noise ratio (SNR) or water-vapor absorption, 189 spectral bands remain. The Salinas dataset, another AVIRIS subset, has a spatial resolution of 512×217 pixels at 3.7 m per pixel.

The HYDICE-Urban dataset consists of 80×100 pixel images captured by the Hyperspectral Digital Imagery Collection Experiment (HYDICE) sensor (Hughes Danbury Optical Systems Inc., Danbury, CT, USA) [76], covering 162 bands from 400 to 2500 nm. The data originate from the US AG Center [77], and bands affected by atmospheric conditions were discarded. In this scene, cars and roofs constitute the anomalies.

Finally, the Arizona dataset introduced by Watson et al. [73] contains five scenes captured by an unmanned aerial vehicle (UAV) equipped with a Pika-L sensor (Resonon Inc., Bozeman, MT, USA) [78]. The data comprise 281 spectral bands with a spatial resolution of 0.1 m, and several bands affected by atmospheric conditions were discarded. Anomalies include vehicles and other foreign objects embedded in the backgrounds of soil, vegetation, and water.

As shown in Table 1, all datasets exhibit severe pixel-level class imbalance, with anomaly pixels typically accounting for less than 1% of the total pixels. The number of anomaly pixels varies significantly across datasets, ranging from as few as 21 pixels (HYDICE-Urban) to over 5000 pixels (Arizona), further highlighting the variability in detection difficulty. Here in the table, the number of bands column shows channels after preprocessing.

### 3.12. Performance Metrics for Class Imbalanced HS-AD

We evaluated the effectiveness of the anomaly detection algorithms using the macro-averaged F1-score (F1-macro), area under the receiver operating characteristic curve (ROC-AUC), area under the precision–recall curve (PR-AUC), and true-positive rate at a fixed false-positive rate (TPR@FPR). Together, these metrics provide a comprehensive assessment under severe class imbalance. In HS-AD, anomalous pixels typically constitute a very small fraction of the scene, making any single metric alone insufficient and potentially misleading for performance evaluation [60,79].

Precision measures the reliability of detected anomalies, while recall quantifies the ability to retrieve rare anomalous pixels. The F1-score is the harmonic mean of precision and recall:

(19) $$F1=2\frac{\mathrm{Precision}\times \mathrm{Recall}}{\mathrm{Precision}+\mathrm{Recall}}\;F1_{\mathrm{macro}}=\frac{1}{C_{\mathrm{cls}}}\sum_{c=1}^{C_{\mathrm{cls}}}F1_{c}$$

where $C_{\mathrm{cls}}$ is the number of classes; here, $C_{\mathrm{cls}}=2$ for anomaly and background.

Throughout this paper, class-macro denotes the unweighted mean over the anomaly and background classes, as in F1-macro above. Dataset-macro denotes the unweighted mean of the dataset-level metric values, so each dataset contributes equally regardless of its number of pixels. Pixel pooling instead concatenates the predictions and labels from all evaluated pixels before computing one metric and therefore gives larger datasets greater influence. Unless a caption states otherwise, repeated-run results are computed for each dataset and run first and are then reported as the mean and standard deviation across runs.

Since the F1-score is threshold-dependent, we compute it across all possible decision thresholds derived from the precision–recall curve and report the maximum value (best F1). The corresponding decision threshold is selected at the point that maximizes the F1-score (computed only on the anomaly class). Note that the optimal threshold is selected independently for each dataset based on its precision–recall curve, reflecting dataset-specific operating conditions under varying levels of class imbalance.

We report F1-macro at this optimal threshold to give equal importance to both anomaly and background classes, mitigating bias toward the dominant background class.

ROC-AUC evaluates the separability between anomalous and background pixels across all thresholds by integrating the true positive rate against the false positive rate, providing a threshold-independent measure of ranking quality under skewed distributions.

Precision–recall-based metrics provide a more informative evaluation under such an imbalance. PR-AUC summarizes the precision–recall trade-off across thresholds and is more informative than ROC-AUC, as it focuses on the performance of the minority (anomaly) class. Unlike ROC-AUC, which can present overly optimistic results under severe class imbalance, PR-AUC explicitly penalizes false positives through precision and therefore provides a more faithful assessment of anomaly detection performance.

The true positive rate at a fixed false-positive rate (TPR@FPR) evaluates detection performance under strict false-alarm constraints by measuring TPR at a predefined FPR. This is particularly important in HS anomaly detection, where even a low false-positive rate can lead to a large number of false alarms, making evaluation at controlled operating points essential. It complements ROC-AUC and PR-AUC by assessing detection capability under practically relevant operating conditions.

Together, these metrics provide complementary insights: F1-macro reflects detection quality at an operating threshold, ROC-AUC measures overall ranking performance, PR-AUC captures performance under class imbalance, and TPR@FPR evaluates effectiveness under practical low-false-alarm constraints. These metrics are particularly suitable for evaluating domain generalization, as they capture both threshold-independent ranking and performance under strict operating conditions across heterogeneous datasets, which is critical for assessing the effectiveness of the proposed Mixture-of-Experts framework.

## 4. Results

We evaluated the performance of the proposed model on seven HS datasets (domains) acquired by different sensors (AVIRIS, HYDICE, Pika-L, and ROSIS-03) and across diverse scenes. We split each dataset individually in a stratified manner based on class labels, allocating 32% for training, 8% for validation, and 60% for testing, thereby preserving the original class imbalance in each domain. Using these splits, the training portions of all or some of the datasets were combined to train the complete neural network end-to-end, including the neural-expert pool, enabling joint learning across domains while maintaining strict separation of the held-out test sets. The test data were not used during training, hyperparameter tuning, or model selection, and were employed exclusively for the final performance evaluation reported in this section.

### 4.1. GMoE-AD Trained on All Datasets

Table 2 presents the performance of GMoE-AD trained on the combined training data from all seven datasets. We report ROC-AUC to measure ranking ability, PR-AUC to assess robustness under class imbalance, F1-macro to evaluate detection quality, and TPR@FPR to capture performance under low false-alarm constraints. For each dataset, pixels are randomly sampled from the validation set to compute the precision–recall curve, and the decision threshold is selected to maximize the F1-score for the anomaly class (binary F1). This threshold is then used to compute the corresponding F1-macro score (Best F1-macro). Together, these metrics provide a comprehensive evaluation under severe class imbalance. Dataset rows are reported as the mean ± standard deviation over five runs. The Average row is the dataset-macro mean, with its standard deviation computed across the seven dataset-row means.

The average best F1-macro score of 0.821±0.094 demonstrates moderate detection quality at an optimal threshold selected on the validation set. Additionally, the model achieves TPR values of 0.712±0.200 at 1% FPR and 0.838±0.164 at 5% FPR, indicating its ability to detect anomalies under low false-alarm constraints, which is critical for practical HS anomaly-detection systems. These 1% and 5% false-positive rates are commonly used operating points in anomaly detection, representing strict and moderately relaxed false-alarm regimes. They allow consistent comparison of models under practically meaningful conditions.

Performance varies across datasets. The model achieves strong results on HYDICE-Urban, San-Diego, and ABU-Urban, with ROC-AUC values exceeding 0.99 and consistently high TPR at low FPR. In contrast, performance degrades on more challenging datasets, such as Arizona and ABU-Beach, as reflected by lower PR-AUC and F1 scores along with higher variance. This suggests that domain shift and scene complexity significantly impact detection performance. Arizona data were collected using a different sensor (Pika-L), resulting in a distinct spectral distribution and reduced alignment with the training domains, which limits effective neural-expert specialization.

**Figure 4:**
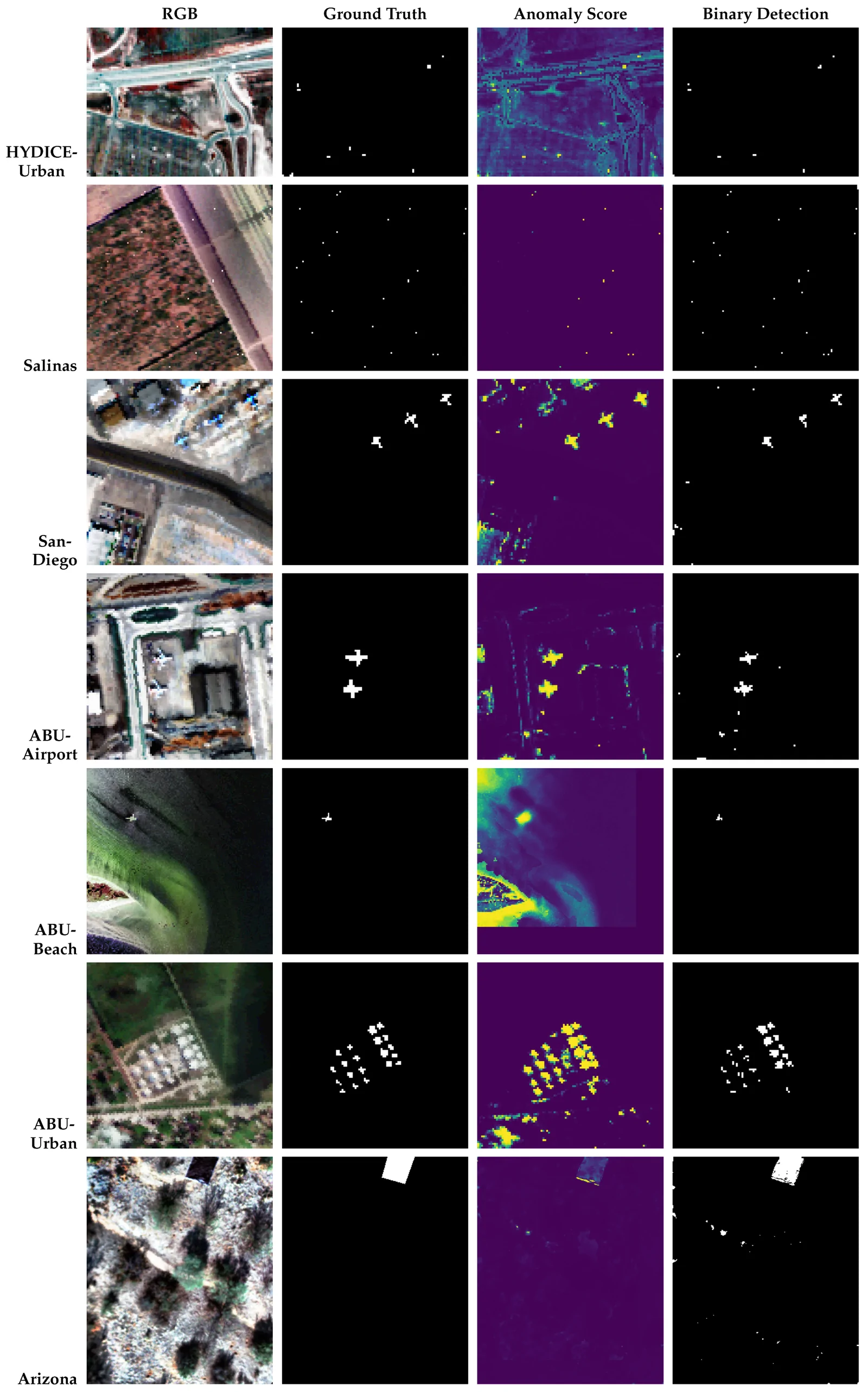
Qualitative results across datasets. Rows ((**top**) to (**bottom**)): HYDICE-Urban (F1 = 0.958), Salinas (F1 = 0.933), San-Diego image 02 (F1 = 0.913), ABU-Airport image 02 (F1 = 0.819), ABU-Beach image 01 (F1 = 0.976), ABU-Urban image 02 (F1 = 0.942), Arizona image 04 (F1 = 0.923). Columns ((**left**) to (**right**)): red–green–blue (RGB) image, ground-truth mask, anomaly score map, and binary detection result. Here, in the ground-truth and binary maps, white denotes anomaly pixels and black denotes background pixels. In the anomaly-score maps, colors progress from dark purple/blue (low anomaly score) to yellow (high anomaly score).

Figure 4 shows qualitative examples of the model’s detection maps. On average, the model achieves a ROC-AUC of 0.943±0.082, indicating strong ranking ability across domains. The relatively lower PR-AUC (0.623±0.191) reflects the difficulty of maintaining precision under extreme class imbalance, where precision–recall characteristics are more sensitive to rare anomalies.

The qualitative maps further illustrate both successful detections and the most challenging background conditions. GMoE-AD is particularly effective when anomalies exhibit clear spectral–spatial contrast relative to the local background, as demonstrated by the compact vehicles and man-made targets. On ABU-Beach, the remaining false responses are concentrated near beach–water transitions, wave or specular patterns, and heterogeneous shoreline regions, while very small or low-contrast objects remain more difficult under extreme class imbalance. Arizona introduces greater spectral variability through its Pika-L sensor and diverse soil, vegetation, and water backgrounds. Even under these conditions, the representative map retains useful anomaly localization, although target-like background regions can increase false responses and weak target contrast can reduce sensitivity. These patterns provide qualitative context for the PR-AUC and TPR@FPR results for ABU-Beach and Arizona in Table 2.

Overall, the all-domain results indicate strong performance across datasets represented during training. We also conducted LODO experiments to evaluate performance on unseen datasets. The higher variability observed on ABU-Beach and Arizona identifies conditions where broader domain coverage and further calibration may improve stability.

### 4.2. Computational Cost and Inference Profile

We assess computational cost at two complementary levels. First, we profile the neural model to quantify its parameter count, arithmetic complexity, memory usage, and neural-stage latency. Second, we report classical base-map generation and neural-stage timings separately. Because these stages were measured on different hardware, their runtimes are not added or treated as a direct cross-system comparison.

#### 4.2.1. Neural Model Profile

The neural profile corresponds to the frozen GMoE-AD top-2 configuration. It includes the HyperSL backbone, soft-gating router, FFNN-AD neural experts, and weighted fusion, while excluding the training-only GRL and domain-classifier branch. The profile also excludes the optional DTA variant. When DTA is activated, it requires additional test-time optimization steps, and its runtime depends on the detected drift level and the adaptation operations selected for a given scene. Arithmetic complexity is reported as multiply–accumulate operations (MACs) and floating-point operations (FLOPs), where the prefix G denotes billions of operations.

The standardized profile was measured on a Rocky Linux compute node equipped with a 64-core CPU, 768 GB RAM, and eight NVIDIA H100 80GB HBM3 GPUs; each profiling job requested and used one GPU. Inference used bfloat16 precision with the Python, PyTorch, and CUDA versions reported in Section 3.7.

Table 3 shows that the model remains compact in parameter count. Complexity is reported both per sample and for the profiled batch so that the result is not dependent on an unstated batch size. The MoE-specific components remain lightweight relative to the shared spectral backbone because the router and neural experts are small feedforward subnetworks. The memory usage nevertheless indicates that the implementation is not optimized for strict onboard real-time deployment, and memory-efficient inference, quantization, or backbone compression remain important directions.

#### 4.2.2. Hardware-Specific Performance and Cost Comparison

**Figure 5:**
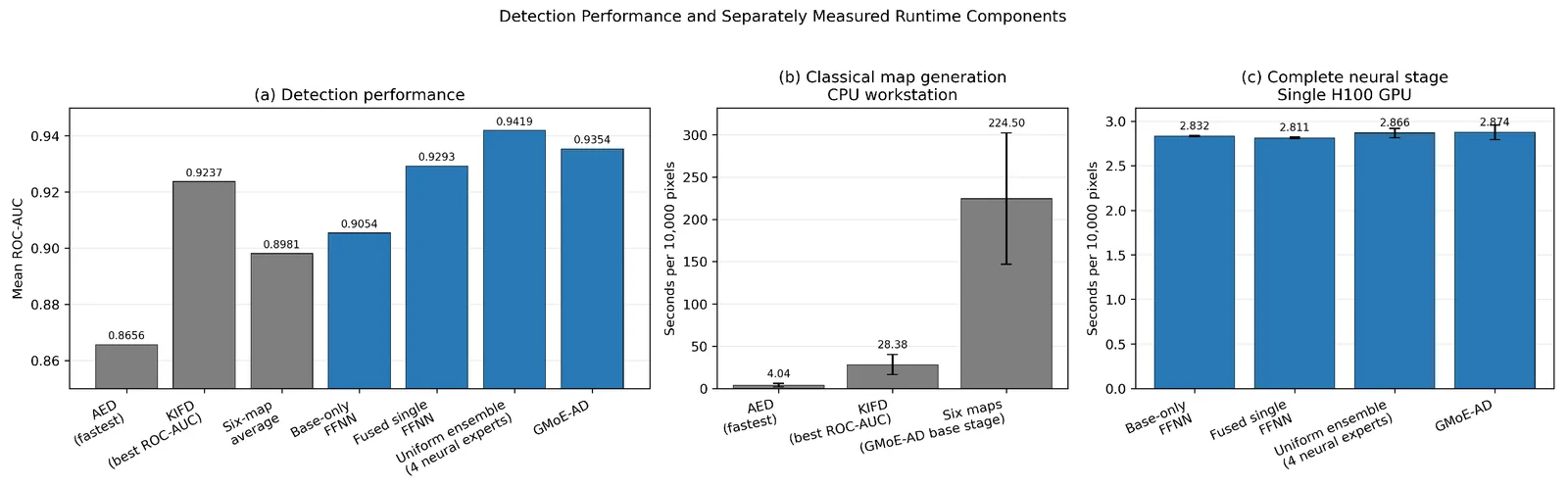
Detection performance and separately measured runtime components per 10,000 pixels. GMoE-AD denotes the complete proposed configuration with learned top-2 neural-expert routing. (**a**) Mean ROC-AUC, including AED as the fastest base detector and KIFD as the base detector with the highest mean ROC-AUC. (**b**) Classical detector-map generation on the CPU workstation; generation of all six maps is the required GMoE-AD base stage. (**c**) Complete neural-stage latency on one H100 GPU, using the two fixed seeds common to all four architectures. Panels (**b**,**c**) use different hardware and measurement scopes and therefore are not added or directly compared.

The comparison in Table 4 and Figure 5 separates detection performance, classical base-map generation, and neural-stage latency. All timing results are normalized to 10,000 pixels. Classical base-detector timings were measured on a workstation running Ubuntu 24.04.4 LTS, equipped with an Intel Core i9-9900K processor (Intel Corporation, Santa Clara, CA, USA; 8 physical cores, 16 logical processors, 3.60 GHz) and 64 GB RAM. The detector implementations used MATLAB R2022b Update 10 (The MathWorks, Inc., Natick, MA, USA) with eight computational threads. The classical benchmark used eight complete repetitions over 22 scenes and generated the six detector maps sequentially. The publicly available MATLAB base-detector implementations were evaluated unmodified on the CPU, as the released source code did not include GPU-enabled implementations (We implemented and validated a GPU version of Fast-KRX on an RTX 2080 Ti. It produced equivalent maps and only 1.07× aggregate speedup (22 scenes; 25 warm repetitions; 1.27× on five Arizona cubes); no general GPU scaling was applied).

For the neural comparison, we use the two fixed seeds (6603 and 1954) for which all four architectures were profiled using one NVIDIA H100 80 GB GPU on a university high-performance computing (HPC) node (Rocky Linux, 64-core CPU, 768 GB RAM, and eight H100 GPUs). Each configuration uses 25 timing repetitions per seed. The complete neural stage includes data loading, host-to-GPU transfer, and synchronized forward computation, but excludes base-map generation. Thus, the classical and neural timings describe separate computational stages and should not be added or compared as measurements of the same operation.

The GMoE-AD ROC-AUC of 0.9354 in Table 4 is the fixed-seed result for the full top-2 configuration (A6), defined in Section 4.7.1. This value differs slightly from the primary five-run ROC-AUC of 0.943 reported in Table 2 because it was obtained using the controlled fixed-seed ablation protocol.

On the CPU workstation, AED is the fastest base detector at 4.04±2.10 s per 10,000 pixels, whereas KIFD, the base detector with the highest mean ROC-AUC, requires 28.38±11.93 s. Sequential generation of all six detector maps, which forms the GMoE-AD base stage, requires 224.50±77.80 s. Thus, on the same CPU workstation, this required stage alone is approximately 55.5× the AED runtime and 7.9× the KIFD runtime. GMoE-AD then requires its neural stage, measured separately on the H100 GPU. In that benchmark, the four neural stages require approximately 2.81–2.87 s. The hardware-controlled H100 measurements show similar neural-stage latency across the single-FFNN and multi-neural-expert architectures; therefore, they do not demonstrate an inference-speed advantage from top-2 routing. The detector maps are amenable to parallel execution after shared preprocessing, but the present classical benchmark uses sequential execution. In the controlled fixed-seed comparison, GMoE-AD achieves a mean ROC-AUC of 0.9354, compared with 0.9237 for KIFD. The difference is more pronounced for metrics emphasizing the rare anomaly class: GMoE-AD achieves a PR-AUC of 0.6355 compared with 0.4125 for KIFD, and TPR at 1% FPR of 0.7120 compared with 0.6331 for KIFD. These results indicate that combining the six detector maps with the learned HyperSL representation primarily improves precision–recall quality and anomaly sensitivity at low false-positive rates. This benefit must be weighed against the additional base-map computation; KIFD remains preferable when computational efficiency is the primary requirement. Among the learned configurations sharing this base-map cost, uniform averaging of four neural experts gives the highest mean ROC-AUC, while the fused single FFNN gives the highest F1-macro and PR-AUC in Table 5.

### 4.3. Leave-One-Dataset-Out Generalization

Table 6 reports the performance of the proposed model under the LODO evaluation protocol. In each experiment, the model is trained on all but one dataset and evaluated exclusively on the held-out dataset, thereby simulating deployment under an unseen-domain scenario. The results are reported as mean ± standard deviation over three repetitions using independently selected random seeds. The same set of imbalance-aware metrics is reported to enable direct comparison with the all-domain training results in Table 2. Since the F1-score is threshold-dependent, we adopt a hold-out strategy to estimate the decision threshold. Specifically, for each dataset, 20% of the pixels are randomly selected to compute the precision–recall curve and determine the optimal threshold that maximizes the F1-score on the anomaly class. This subset is not used for metric reporting. The remaining 80% of the pixels are used exclusively for evaluation, where F1-macro and other metrics are computed using the selected threshold. The process is repeated across runs with different random splits, and results are reported as mean ± standard deviation. The performance exhibits substantially higher variability than under all-domain training experiments, with F1-macro scores ranging from 0.650 to 0.888, indicating that this setting is significantly more challenging. Compared to the all-domain training results in Table 2, where the average F1-macro reaches 0.821, the LODO setup leads to noticeable degradation on several datasets, highlighting the sensitivity of HS-AD models to domain shift when the target dataset is entirely excluded from training.

The largest performance degradation occurs on the Arizona dataset, collected with the Pika-L sensor. Because no other Pika-L dataset is available during training, the model lacks sensor-consistent representations, leading to a sharp drop in F1-macro (0.650±0.014). This result underscores the importance of sensor diversity for cross-domain generalization, as performance degrades when the training domains do not provide sensor-consistent examples for neural-expert routing.

A similar, though less severe, degradation is observed for ABU-Beach. This dataset includes imagery from multiple sensors, increasing intra-dataset heterogeneity and making it difficult for the model to learn a coherent anomaly representation when the dataset is fully excluded during training.

In contrast, performance on HYDICE-Urban remains comparatively stable despite the absence of other HYDICE datasets in training. This can be attributed to scene-level similarity with ABU-Urban, which allows knowledge transfer across datasets even when sensor characteristics differ.

For datasets collected using AVIRIS (e.g., San-Diego, ABU-Airport, ABU-Urban, and Salinas), the model achieves consistently higher performance. The availability of multiple AVIRIS datasets may support collaborative learning among neural experts, mitigating domain shift and improving the robustness of anomaly detection in the LODO setting.

**Figure 6:**
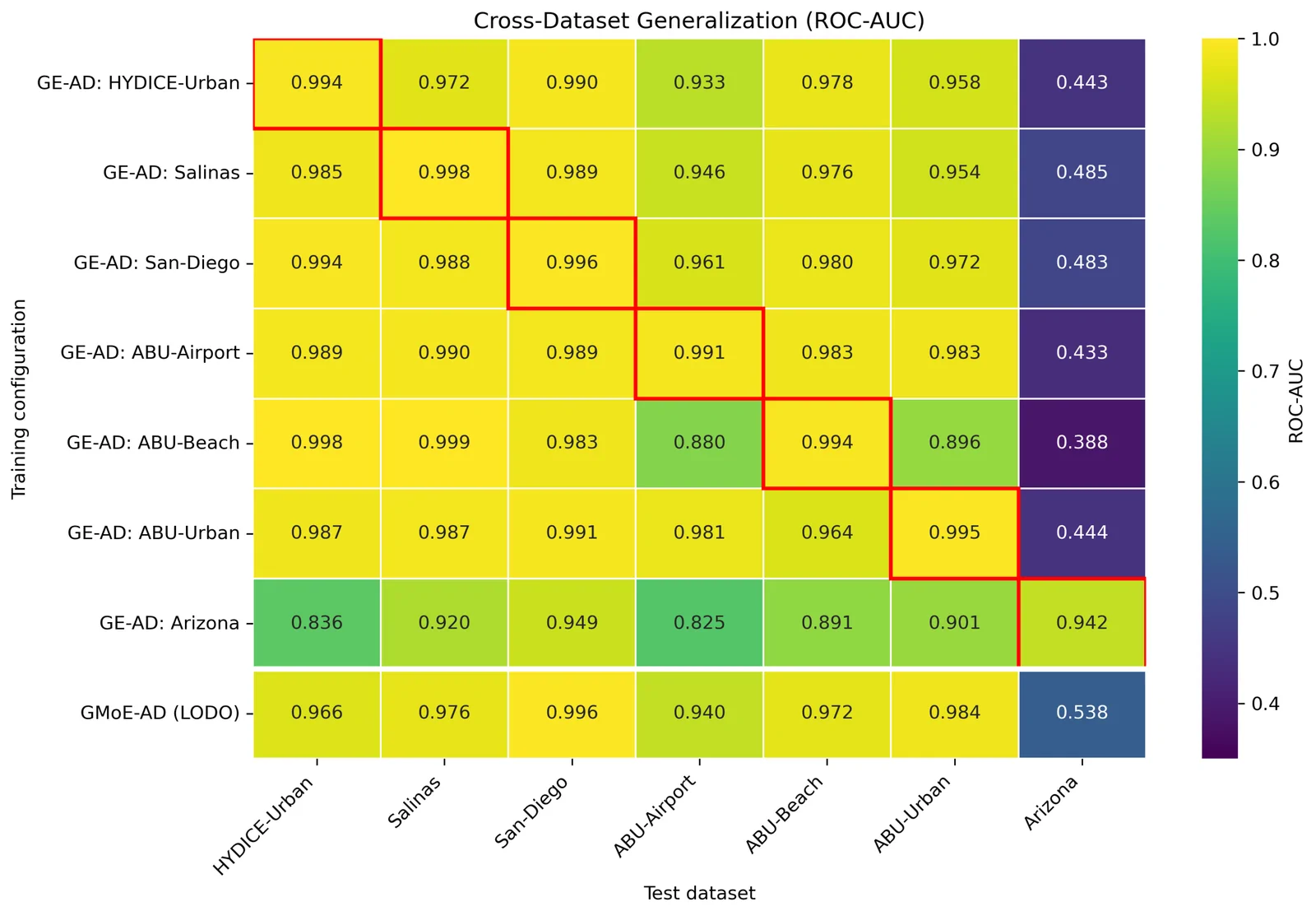
Cross-dataset ROC-AUC of single-domain GE-AD models and LODO GMoE-AD. Rows 1–7 denote the dataset used to train each GE-AD model, and columns denote the test dataset; the red diagonal marks matched training and testing datasets. In the final row, each GMoE-AD value is the across-run mean reported in Table 6 for a model trained without that test dataset.

These results further suggest that the Mixture-of-Experts framework benefits from exposure to diverse domains during training, while performance degrades when domain-specific characteristics are entirely absent. Figure 6 places the mean ROC-AUC values from this LODO evaluation alongside the cross-dataset behavior of the single-domain GE-AD models.

### 4.4. Effect of Drift-Aware Test-Time Adaptation

We evaluate DTA separately as an optional adaptive variant of GMoE-AD under both the all-domain training and LODO protocols. Table 7 reports dataset-macro averages. Table 8 reports the corresponding dataset-level changes in the two ranking metrics. The all-domain comparison uses three paired fixed-seed evaluations (8264, 6603, and 1954), while the LODO comparison uses the three paired repetitions described in Section 4.3. The frozen model remains the primary configuration; DTA was disabled for the primary results and component ablations and was enabled only for the comparisons in this subsection.

DTA provides a small improvement in ranking-based metrics under all-domain training, increasing dataset-macro ROC-AUC and slightly improving PR-AUC and TPR at low false-positive rates. However, the F1-based metrics decrease slightly, indicating that adaptation can alter score calibration under some operating thresholds. At the dataset level, DTA is most beneficial for ABU-Beach, where heterogeneous beach–water backgrounds and sensor variability create stronger distribution drift. In contrast, Arizona shows improved ranking but degraded precision-sensitive behavior.

Under LODO evaluation, DTA reduces all six macro-averaged metrics. ROC-AUC decreases from 0.910 to 0.886, and F1-macro decreases from 0.773 to 0.752. DTA improves both ROC-AUC and PR-AUC only for ABU-Urban; it improves PR-AUC but not ROC-AUC for Salinas and HYDICE-Urban. The largest ROC-AUC reductions occur for ABU-Beach (−0.086) and Arizona (−0.041). Thus, the fixed adaptation policy does not transfer reliably when the entire target dataset is absent during training, and frozen inference remains the preferred configuration under the evaluated LODO protocol. Taken together, the two evaluations characterize DTA as an optional, evaluation-dependent adaptation mechanism rather than a universally beneficial replacement for frozen inference.

In the all-domain evaluation, the adaptation diagnostics contain 3096 evaluated minibatches across all 22 scenes. Every scene and minibatch activated at least one adaptation operation: 2385 minibatches (77.03%) updated BN statistics together with BN and LN affine parameters, while 711 minibatches (22.97%) updated BN statistics and BN affine parameters without LN adaptation. Thus, under the fixed thresholds used here, the policy controlled adaptation intensity but did not select the no-adaptation branch. This evaluation therefore does not constitute a sensitivity study of the DTA thresholds.

### 4.5. Comparison with Base Methods

Table 9 presents a quantitative comparison between the proposed method and base HS-AD and unmixing-AD methods. Because most of these methods are unsupervised and produce continuous anomaly scores without a canonical (i.e., method-intrinsic and dataset-independent) decision threshold, threshold-dependent metrics such as the F1-score would require additional assumptions and heuristic tuning. PR-AUC does not require a decision threshold, but it was unavailable for some historical base-method outputs. ROC-AUC therefore provides the common threshold-independent comparison and evaluates the ranking quality of anomaly scores across all possible operating points. While not strictly comparable due to supervision differences, we adopt ROC-AUC as the primary evaluation metric here, as it is reproducible and assumption-free, and it allows a comparison with prior work.

Recent transfer-enhanced and continual cross-domain HS-AD methods provide important context for this study [22,23,24]. These methods are commonly evaluated under single-dataset, predefined source–target, or sequential-learning protocols, whereas GMoE-AD is evaluated without access to the target-dataset identity during inference. Because these protocols measure different deployment settings, their reported numerical results are not directly comparable. We therefore report reproducible comparisons with base detectors evaluated on the same datasets and use the LODO experiments in Section 4.3 to evaluate generalization when the target dataset is excluded from training.

Table 9 distinguishes all-domain training from strict LODO evaluation. All-domain GMoE-AD achieves the highest average ROC-AUC (0.943), followed by KIFD (0.924). Under LODO evaluation, however, GMoE-AD averages 0.910, while KIFD retains the higher average of 0.924. KIFD performs better on HYDICE-Urban, Salinas, ABU-Airport, and Arizona, whereas LODO GMoE-AD performs better on San-Diego, ABU-Beach, and ABU-Urban. Thus, KIFD provides the stronger simple choice when strict unseen-domain mean ROC-AUC and computational cost are the primary criteria. GMoE-AD instead provides a unified learned framework for feature fusion, domain-adversarial representation learning, neural-expert routing, and optional test-time adaptation; these capabilities do not guarantee superior ROC-AUC when the target domain is entirely absent from training.

In the all-domain training setting, the proposed method demonstrates substantial improvement on the Arizona dataset, achieving a ROC-AUC of 0.748 compared with 0.609 for KIFD, 0.345 for AED, and 0.338 for LSUNRSORAD. As Arizona represents a more challenging and distinct domain, this result suggests that including representative Arizona data improves performance on this distinct domain. While most baseline methods exhibit a sharp performance drop on this dataset, the proposed method maintains comparatively stable performance, highlighting the benefit of including representative Arizona data during training. When Arizona is excluded under LODO, GMoE-AD decreases to 0.538; therefore, this all-domain result should not be interpreted as superiority on an entirely unseen Arizona domain.

### 4.6. Comparison with Single-Domain GE-AD Models

To compare cross-dataset ranking performance without introducing differences in decision-threshold selection, Figure 6 uses ROC-AUC for both model families. Each of the first seven rows represents a single-domain GE-AD model trained on the dataset named in the row and evaluated on every test dataset. The final row reports GMoE-AD under the LODO protocol of Section 4.3; consequently, each entry in that row is obtained from a model trained without the corresponding test dataset. The red diagonal identifies the matched training–test condition for each single-domain GE-AD model and is included as a reference rather than as an unseen-domain result.

The single-domain GE-AD models transfer well among most of the six public datasets, with many off-diagonal ROC-AUC values above 0.95. This indicates that several of these datasets share sufficiently similar anomaly-ranking cues for a model trained on one dataset to remain effective on another. The principal exception is Arizona: GE-AD models trained on any of the six public datasets obtain only 0.388–0.485 ROC-AUC on Arizona. Conversely, the Arizona-trained GE-AD model performs well in its matched single-domain condition (0.942) but is less consistent on several public datasets, particularly HYDICE-Urban and ABU-Airport.

GMoE-AD achieves strong LODO ROC-AUC on all six public datasets (0.940–0.996) and improves unseen-Arizona ROC-AUC to 0.538 relative to every GE-AD model trained on a different dataset. However, it remains below the Arizona-trained GE-AD model because the latter has access to matched Pika-L and desert-scene training data. Thus, Figure 6 shows that GMoE-AD generalizes well to the six held-out public datasets. When Arizona is excluded from training, GMoE-AD also outperforms every single-domain GE-AD model trained on another dataset. Nevertheless, its ROC-AUC remains below that of the GE-AD model trained directly on Arizona, indicating that training on the other six datasets cannot fully compensate for the absence of sensor- and scene-matched Arizona examples.

### 4.7. Component and Loss Analyses

We evaluate the contributions of the model architecture, neural experts, base detectors, routing losses, and domain-adversarial training through a series of controlled ablation and diagnostic experiments.

#### 4.7.1. Architecture and Routing Ablation

The architecture ablation isolates the contributions of feature fusion, multiple neural experts, learned gating, and sparse routing. All configurations were evaluated using the same datasets, data splits, training procedure, and three random seeds. The first three configurations use a single FFNN classifier: A0 uses only HyperSL features, A1 uses only the six base-detector outputs, and A2 combines both feature sources. Comparing A0 and A1 with A2 measures the benefit of feature fusion, while A2 provides the single-network reference for evaluating the multi-expert configurations.

Configurations A3–A6 use four FFNN neural experts with the fused HyperSL and base-detector representation. A3 assigns equal weight to every neural expert and therefore isolates the benefit of multiple neural experts without learned gating. A4 uses learned dense routing, with all four neural experts contributing to each prediction. A5 and A6 apply learned sparse routing and retain the highest-weighted one and two neural experts, respectively. These configurations determine whether learned gating improves upon uniform averaging and whether sparse routing preserves the benefits of combining multiple neural experts.

**Figure 7:**
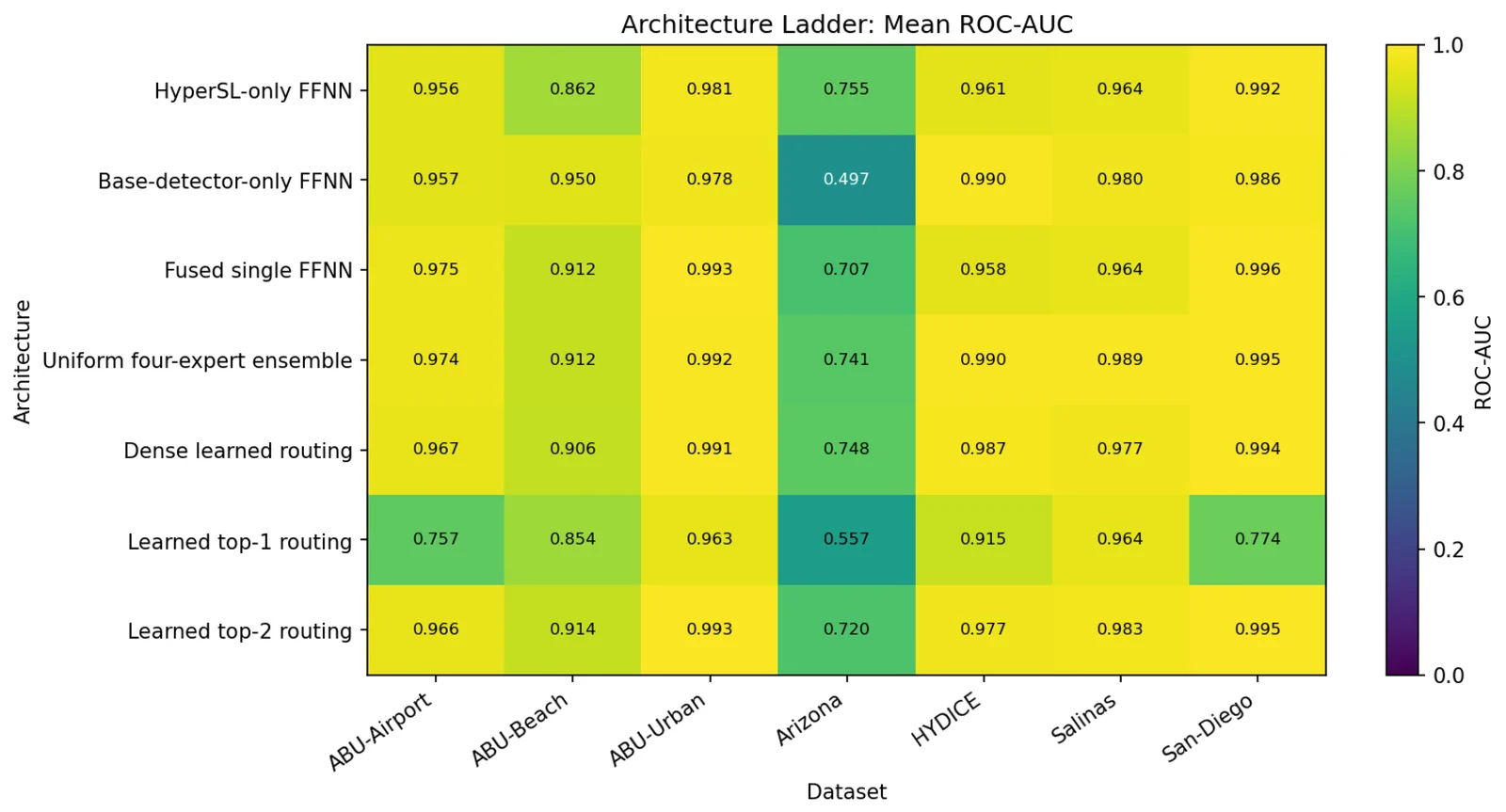
Dataset-wise ROC-AUC for the architecture ablation. The configurations are defined in Table 5.

Fusing HyperSL and base-detector inputs in A2 gives the highest F1-macro and PR-AUC. Uniform averaging in A3 gives the highest mean ROC-AUC, showing that complementary neural-expert predictions are useful without adaptive routing. Learned dense and top-2 routing remain competitive, whereas top-1 routing causes a substantial reduction. Thus, top-2 routing is a performance–sparsity compromise rather than the universally highest-scoring architecture. No configuration is uniformly optimal across datasets (Figure 7).

#### 4.7.2. Neural Expert Specialization

**Figure 8:**
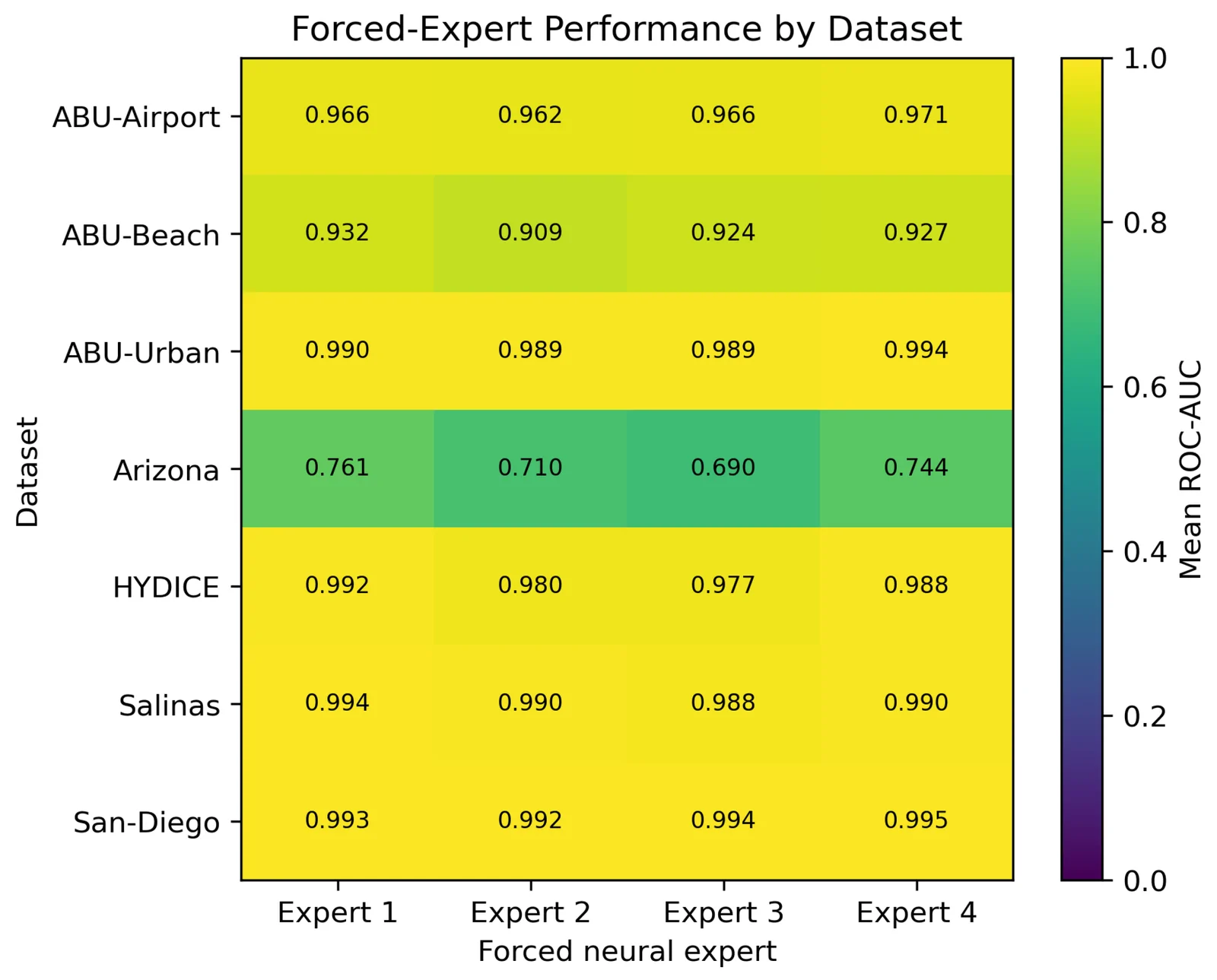
ROC-AUC when each neural expert is evaluated separately under the full top-2 configuration, averaged across three fixed random seeds. Each column reports inference restricted to one jointly trained neural expert; this is a diagnostic rather than a causal architecture ablation.

Figure 8 shows complementary but not uniformly strong specialization. Neural Expert 1 is strongest on ABU-Beach, Arizona, HYDICE-Urban, and Salinas, whereas Neural Expert 4 is strongest on ABU-Airport, ABU-Urban, and San-Diego. The largest separation occurs on Arizona, where mean ROC-AUC ranges from 0.690 to 0.761; spreads are below 0.01 on several AVIRIS datasets. Latent sensor and scene similarities are a possible explanation, but this diagnostic does not establish a causal assignment.

#### 4.7.3. Routing Diagnostics

Evaluating each jointly trained neural expert separately describes its behavior in isolation. We next examine the complementary question of how the router combines these neural experts under the reference top-2 configuration. Table 10 summarizes routing statistics from three fixed-seed evaluations at three stages: the raw dense probabilities before top-*k* selection, the post-temperature dense probabilities, and the effective probabilities after top-*k* masking. The dense probabilities have high normalized entropy and very low Kullback–Leibler (KL) divergence to a uniform distribution, indicating that probability mass is distributed across the neural experts before sparsification. After top-*k* selection, the effective routing becomes sparse as expected because only two of four neural experts are retained for each sample.

The top-*k* analysis indicates soft specialization rather than a hard fixed assignment of one neural expert to each domain. Dataset-dependent selection tendencies are present, but the gate does not simply select the independently strongest neural expert for each dataset. The routing-loss results below show that load balancing encourages the use of all neural experts, while the other routing regularizers help the router express clearer preferences among them. However, no single neural expert dominates all datasets.

#### 4.7.4. Base-Detector Removal

**Figure 9:**
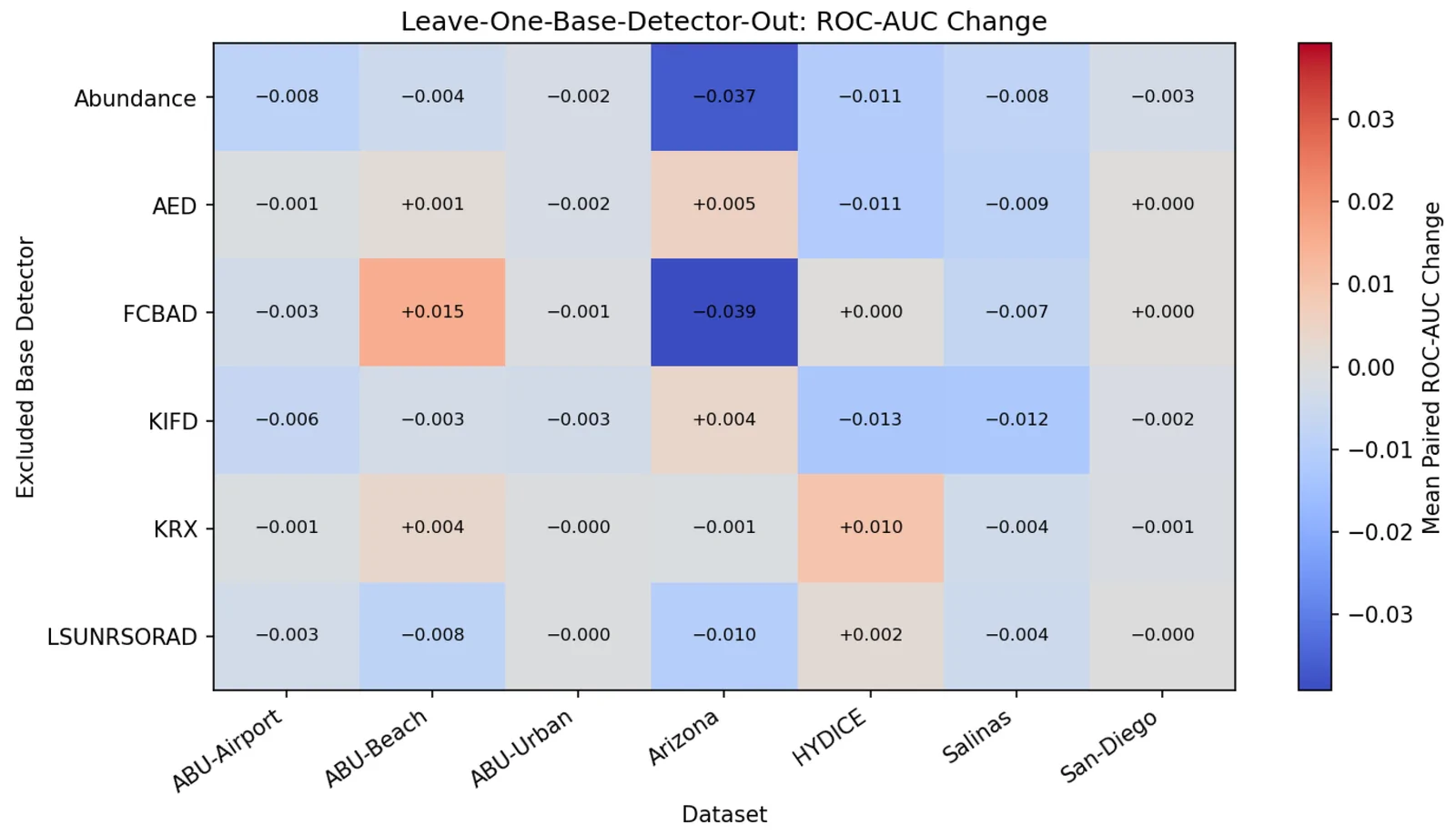
Dataset-wise paired ROC-AUC change after removing one base detector. Negative values indicate a positive contribution by the removed detector.

Table 11 shows that abundance gives the largest average ROC-AUC contribution, while KIFD gives the largest F1-macro and PR-AUC contribution. FCBAD has a mixed effect: its removal lowers ROC-AUC but improves the threshold-sensitive metrics. Removing KRX leaves the averages essentially unchanged, indicating partial redundancy rather than a uniformly harmful contribution. The effects are strongly dataset-dependent, as shown in Figure 9.

#### 4.7.5. Routing-Loss Ablation

Table 12 shows that removing the routing losses generally improves average detection metrics, so they are not interpreted as universally beneficial for predictive accuracy. The corresponding routing statistics in Table 13 show that removing load balancing increases selection concentration and neural-expert under-utilization. Removing all routing regularization instead produces nearly uniform pre-top-*k* probabilities, although top-2 ranking still favors a subset of neural experts. The losses therefore shape utilization balance and gate differentiation, with a trade-off against average detection scores.

#### 4.7.6. Domain-Adversarial Ablation

In the ablation study, we remove the domain classifier head, effectively reducing the model to a standalone anomaly detector, and evaluate the impact. Because the GRL operates only through the domain-classifier branch, removing the domain-classifier head also removes the GRL and the associated domain-adversarial loss ($L_{grl}$). In the no-GRL variant, the domain-classifier head remains active and is trained using standard multi-class cross-entropy, but its gradients are not reversed before reaching the shared backbone. Table 14 shows that removing the domain classifier degrades F1-macro performance relative to the full model (with GRL and domain classifier, 0.821±0.094 in Table 2), indicating that the auxiliary task improves anomaly detection on the evaluated all-domain test sets. This ablation does not by itself establish improved unseen-dataset generalization.

As shown in Table 14, removing the GRL degrades performance relative to the full model, highlighting the importance of adversarial domain alignment for learning domain-robust representations. Overall, these results show that both the auxiliary domain classification task and adversarial training improve the measured all-domain anomaly-detection performance.

## 5. Discussion

Table 2 reports dataset-macro F1-macro (0.821±0.094), PR-AUC (0.623±0.191), and ROC-AUC (0.943±0.082), where these standard deviations describe variation among datasets rather than variation among runs. The results show strong average ranking performance but also substantial dataset dependence, particularly for PR-AUC. Comparisons with the base methods (Table 9) show that all-domain GMoE-AD achieves the highest average ROC-AUC, although individual base methods remain stronger on some datasets. This suggests that combining complementary anomaly cues improves average ranking performance across heterogeneous domains, although some base methods perform better on individual datasets.

The controlled component analyses qualify this aggregate comparison. Feature fusion gives the strongest F1-macro and PR-AUC, while uniform averaging of four neural experts gives the highest mean ROC-AUC (Table 5). Learned top-2 routing therefore should be viewed as a sparse performance compromise rather than as uniformly superior to every simpler alternative. Similarly, the base-detector removal results (Table 11) show complementary but nonuniform contributions: abundance is most important to average ROC-AUC, KIFD contributes most to F1-macro and PR-AUC, and KRX is largely redundant on average.

We formulate the problem as a multi-task learning framework by incorporating domain classification as an auxiliary objective. The ablation study confirms its positive contribution to anomaly detection performance (see Table 14). This result indicates that the auxiliary task improves the measured all-domain anomaly-detection performance; its unseen-dataset effect is not isolated by this ablation.

### 5.1. Impact of MoE on Anomaly Detection Metrics

GMoE-AD does not always match the reference performance of single-domain models trained and evaluated on the same dataset; this gap is expected, given the substantially more challenging evaluation setting. In contrast to prior GE-AD models that are trained for a fixed dataset, our approach must infer neural-expert weights from the input through MoE routing, introducing additional sources of error. Importantly, despite this constraint, GMoE-AD maintains competitive performance across heterogeneous datasets without retraining, highlighting its robustness and practical applicability in realistic deployment scenarios where domain labels are unavailable.

The neural-expert diagnostics provide a more specific interpretation. When each neural expert is evaluated separately, performance varies most on Arizona and less on several AVIRIS datasets, while the best neural expert changes across datasets (Figure 8). This supports partial, dataset-dependent specialization rather than a strict fixed partition assigning one neural expert to each domain. Shared sensors or related scene content may contribute to latent similarities learned by the network, but the present experiments do not isolate sensor and scene effects causally. The routing-loss ablation also separates predictive accuracy from routing behavior. Removing the regularizers improves the average detection metrics but produces either greater selection concentration and neural-expert under-utilization or nearly uniform, weakly differentiated gate probabilities. The auxiliary losses therefore shape the intended routing behavior without consistently improving detection scores.

The efficiency comparison reports classical base-map generation and neural-stage latency separately because they were measured on different hardware (Table 4). On a common H100 GPU, the four neural architectures have similar neural-stage latency, so sparse top-2 routing does not provide a measurable neural-stage speed advantage in the present implementation. The separately measured workstation results show that sequential base-map generation is substantial. Parallel base-map computation, backbone compression, and reduced-detector variants remain direct opportunities for lowering deployment latency.

### 5.2. Generalizability

The LODO results reveal pronounced variability in performance, highlighting the vulnerability of HS-AD models to domain shift when the target dataset is completely unseen during training (see Table 6). Severe performance degradation on the Arizona dataset suggests the critical role of sensor diversity, as the absence of Pika-L data prevents learning sensor-consistent representations. In contrast, more stable performance on datasets with similar scenes or shared sensor characteristics indicates that both sensor-level and scene-level similarity facilitate cross-domain knowledge transfer. Overall, these findings emphasize that robust generalization in HS-AD depends strongly on domain diversity and inter-dataset complementarity.

The comparison in Table 9 also establishes an important boundary of the proposed approach. Under the strict LODO protocol, KIFD achieves a slightly higher mean ROC-AUC than GMoE-AD (0.924 versus 0.910) while requiring substantially less computation. GMoE-AD, however, achieves a higher PR-AUC under LODO evaluation (0.505 versus 0.412), indicating better precision–recall performance for the rare anomaly class. Thus, KIFD remains the stronger choice when unseen-domain ROC-AUC and computational efficiency are the primary requirements, whereas GMoE-AD provides an alternative when precision–recall performance is prioritized.

### 5.3. Environmental and Domain Variability

In practical hyperspectral sensing, distribution shifts arise from multiple interacting factors, including sensor response, scene composition, atmospheric conditions, illumination, acquisition geometry, and temporal changes in the sensing environment. The proposed framework does not explicitly disentangle or quantify the individual contribution of each factor. Instead, these factors are implicitly represented within the dataset-level domain distributions observed during training.

Consequently, GMoE-AD is designed to learn domain-robust representations and adaptive neural-expert routing that responds to the combined effects of heterogeneous acquisition conditions rather than to isolated physical causes. This design reflects realistic deployment scenarios in which explicit metadata describing the sensor, atmosphere, or environmental conditions may be incomplete or unavailable during inference. The LODO experiments further show that when an important source of variability is absent from training, such as the Pika-L sensor in the Arizona experiment, performance can degrade. This indicates that the model benefits from broad domain coverage during training and that sensor- and scene-level diversity are important for reliable generalization.

Further separating the effects of sensor characteristics, scene composition, and atmospheric conditions would benefit from controlled datasets in which these factors can be varied independently. As such datasets remain limited for HS-AD, the present study evaluates their combined influence through dataset-level cross-domain experiments. Future work could extend this analysis through factor-specific domain attribution, explicit environmental modeling, and carefully validated continual or test-time adaptation under changing sensing conditions.

### 5.4. Training- and Test-Time Adaptation

We observe in Table 14 that, compared with the adversarial setup, removing GRL results in a decrease in anomaly detection performance (F1-macro score drops from 0.821 to 0.761), indicating that adversarial domain adaptation during training is beneficial.

The optional DTA evaluation reveals a different trade-off (Table 7). DTA improves average ROC-AUC, PR-AUC, and TPR at low false-positive rates, but slightly reduces F1-macro, and its effects vary by dataset. Moreover, all 22 scenes and all 3,096 evaluated minibatches activate some form of adaptation under the fixed thresholds. The policy therefore controls adaptation intensity in this experiment, particularly whether LN affine parameters are updated, but does not demonstrate reliable selection between adaptation and no adaptation. Threshold sensitivity and validation of the no-adaptation branch remain subjects for future work.

## 6. Conclusions

We propose a unified MoE framework for hyperspectral anomaly detection that extends ensemble-based methods to cross-domain settings. The framework fuses the outputs of six unsupervised base detectors with representations from a pretrained HyperSL foundation model, combines four FFNN neural experts through learned top-2 routing, and applies gradient-reversal-based domain-adversarial training. Replacing the Random Forest component in GE-AD with trainable feedforward neural experts enables joint end-to-end optimization. A drift-aware test-time adaptation (DTA) variant is evaluated separately from the primary frozen model.

Evaluated on seven real-world hyperspectral datasets acquired from four different sensors, the proposed model achieves an ROC-AUC of 0.943±0.082, PR-AUC of 0.623±0.191, and F1-macro of 0.821±0.094 (mean ± standard deviation across datasets, averaged over five runs). Across the seven LODO evaluations, in which each test dataset is excluded from training, average performance decreases to an ROC-AUC of 0.910, PR-AUC of 0.505, and F1-macro of 0.773, reflecting the increased difficulty of generalizing across sensors and scene conditions. Although this performance may lag behind methods trained and tested on the same dataset, it provides a more realistic assessment of performance in operational remote sensing scenarios, where prior knowledge of the target domain is unavailable. The optional DTA variant increases mean ROC-AUC from 0.938 to 0.953 but reduces F1-macro from 0.822 to 0.815. Its benefits are therefore metric- and dataset-dependent rather than uniform.

Overall, the results demonstrate that the proposed ensemble learning framework improves robustness to sensor and scene variability and supports anomaly detection under distribution shifts. These findings establish the proposed approach as a step toward generalized hyperspectral anomaly detection in real-world settings, where training and deployment conditions differ. The component analyses show that the main benefits are not uniform across mechanisms: feature fusion gives the strongest F1-macro and PR-AUC, uniform neural-expert averaging gives the highest mean ROC-AUC, and top-2 routing provides sparse neural-expert collaboration without the severe degradation observed for top-1 routing. Base-detector and routing-loss effects are also dataset- and metric-dependent. The hardware-specific timing profiles show similar neural-stage latency for the single-FFNN and multi-neural-expert architectures, while sequential base-map generation remains a substantial separately measured cost.

Future work will focus on improving routing diversity and specialization of the neural expert subnetworks, reducing or parallelizing base-detector computation, developing mechanisms for unsupervised domain discovery, explicitly modeling the separate effects of sensor characteristics, atmospheric conditions, and scene variability, and evaluating DTA threshold sensitivity and the reliability of no-adaptation selection under evolving sensing environments.

## Figures and Tables

**Table 1:** Summary statistics of the hyperspectral datasets used in this study, including spatial resolution, spectral dimensionality, and pixel-level class distribution. The table highlights the severe class imbalance, where anomaly pixels constitute only a small fraction of the scene across datasets. Here, # denotes the number of bands or pixels.

Dataset	File Name	Spatial Size	# Bands	# Background Pixels	# Anomaly Pixels	Anomaly %	Imbalance Ratio (B:A)	Sensor
HYDICE-Urban	HYDICE-urban	(80, 100)	162	7979	21	0.263%	379:1	HYDICE
Salinas	Salinas	(120, 120)	202	14,375	25	0.174%	575:1	AVIRIS
San-Diego	SanDiego-01	(100, 100)	189	9866	134	1.340%	73:1	AVIRIS
	SanDiego-02	(100, 100)	189	9942	58	0.580%	171:1	
ABU-Airport	abu-airport-1	(100, 100)	205	9856	144	1.440%	68:1	AVIRIS
	abu-airport-2	(100, 100)	205	9913	87	0.870%	113:1	
	abu-airport-3	(100, 100)	205	9830	170	1.700%	57:1	
	abu-airport-4	(100, 100)	191	9940	60	0.600%	165:1	
ABU-Beach	abu-beach-1	(150, 150)	101	22,481	19	0.084%	1183:1	AVIRIS
	abu-beach-2	(100, 100)	65	9798	202	2.020%	48:1	
	abu-beach-3	(100, 100)	187	9989	11	0.110%	908:1	
	abu-beach-4	(150, 150)	97	22,432	68	0.302%	329:1	ROSIS-03
ABU-Urban	abu-urban-1	(100, 100)	174	9933	67	0.670%	148:1	AVIRIS
	abu-urban-2	(100, 100)	162	9845	155	1.550%	63:1	
	abu-urban-3	(100, 100)	191	9948	52	0.520%	191:1	
	abu-urban-4	(100, 100)	205	9728	272	2.720%	35:1	
	abu-urban-5	(100, 100)	205	9768	232	2.320%	42:1	
Arizona	Arizona image 01	(780, 291)	252	220,777	6203	2.733%	35:1	Pika-L
	Arizona image 02	(498, 301)	253	146,399	3499	2.334%	41:1	
	Arizona image 03	(849, 291)	259	235,347	11,712	4.741%	20:1	
	Arizona image 04	(358, 293)	253	103,329	1565	1.492%	66:1	
	Arizona image 05	(448, 275)	256	119,618	3582	2.907%	33:1	

**Table 2:** Quantitative performance of the proposed model under all-domain training. Dataset rows are mean ± standard deviation over five runs. In the Average row, each value is the dataset-macro mean and its standard deviation describes variation among the seven dataset-row means; ↑ indicates that higher values are better.

Dataset	ROC-AUC ↑	PR-AUC ↑	Best F1-Macro ↑	TPR@1% FPR ↑	TPR@5% FPR ↑
HYDICE-Urban	0.991±0.010	0.824±0.061	0.925±0.025	0.872±0.096	0.949±0.073
Salinas	0.947±0.027	0.801±0.040	0.913±0.014	0.844±0.031	0.844±0.031
San-Diego	0.997±0.001	0.783±0.026	0.890±0.009	0.950±0.015	0.987±0.005
ABU-Airport	0.956±0.037	0.467±0.053	0.752±0.017	0.573±0.052	0.857±0.093
ABU-Beach	0.973±0.010	0.420±0.113	0.736±0.057	0.574±0.118	0.786±0.096
ABU-Urban	0.992±0.001	0.726±0.022	0.868±0.007	0.822±0.019	0.968±0.010
Arizona	0.748±0.151	0.337±0.092	0.666±0.090	0.351±0.103	0.473±0.153
Average	0.943±0.082	0.623±0.191	0.821±0.094	0.712±0.200	0.838±0.164

**Table 3:** Standardized single-H100 neural profile of GMoE-AD without DTA. Inference complexity and memory are measured for the frozen model. Per-sample complexity is obtained by normalizing profiler totals for an inference batch of 1024 samples. Neural inference forward time excludes data loading and host-to-GPU transfer, whereas the complete neural inference stage includes both operations. Inference timing values are mean ± standard deviation over 25 repetitions for each of three seeds. Training time is reported separately for one complete epoch comprising 629,660 samples in 621 batches of 1024.

Quantity	Value
Total parameters	1.98 M
Trainable parameters	1.85 M
Inference MACs per sample	0.3166 GMACs
Inference FLOPs per sample	0.6351 GFLOPs
Inference MACs per batch (1024 samples)	324.21 GMACs
Inference FLOPs per batch (1024 samples)	650.30 GFLOPs
Neural inference forward per 10,000 pixels	0.2114±0.0004 s
Complete neural inference stage per 10,000 pixels	2.850±0.075 s
Training time per complete epoch	24.10 s
Peak inference GPU memory	7020.57 MiB

**Table 4:** Detection performance and separately measured runtime components per 10,000 pixels. Performance uses the standardized classical evaluation or the three-seed architecture study in Section 4.7.1. A1 is the base-detector-only FFNN, A2 is the fused (base-detectors and HyperSL) single FFNN, A3 is the uniform four-neural-expert ensemble, and A6 is GMoE-AD with learned top-2 routing. Classical map-generation times were measured on the CPU workstation. Neural-stage times were measured on one H100 GPU using the two common hardware-controlled seeds and exclude base-map generation. The two timing scopes are not added or directly compared.

Method	Mean ROC-AUC	Measured Component	Time (s)
AED (fastest base detector)	0.8656	Detector-map generation	4.04±2.10
KIFD (highest base-detector ROC-AUC)	0.9237	Detector-map generation	28.38±11.93
Unweighted six-map average	0.8981	GMoE-AD six-map base stage	224.50±77.80
Base-detector-only FFNN (A1)	0.9054	Complete neural stage	2.8318±0.0081
Fused single FFNN (A2)	0.9293	Complete neural stage	2.8108±0.0089
Uniform four neural-expert ensemble (A3)	0.9419	Complete neural stage	2.8660±0.0527
GMoE-AD, learned top-2 routing (A6)	0.9354	Complete neural stage	2.8738±0.0816

**Table 5:** Macro-dataset architecture ablation averaged over three seeds. A0 is the HyperSL-only FFNN, A4 uses learned dense routing, and A5 uses learned top-1 routing. Configurations A1–A3 and A6 are defined in Table 4. Bold indicates the best result in each metric column.

Configuration	F1-Macro	ROC-AUC	PR-AUC
HyperSL-only FFNN (A0)	0.7867	0.9243	0.5525
Base-detector-only FFNN (A1)	0.7493	0.9054	0.4531
Fused single FFNN (A2)	**0.8311**	0.9293	**0.6577**
Uniform four neural-expert ensemble (A3)	0.8105	**0.9419**	0.6052
Dense learned routing (A4)	0.8178	0.9386	0.6096
Learned top-1 routing (A5)	0.6066	0.8263	0.3314
GMoE-AD, learned top-2 routing (A6)	0.8231	0.9354	0.6355

**Table 6:** Performance of GMoE-AD when one dataset is excluded completely during training. Dataset rows are mean ± standard deviation over three repetitions. The Average row reports the dataset-macro mean of the seven dataset-row means; ↑ indicates that higher values are better.

Dataset	ROC-AUC ↑	PR-AUC ↑	Best F1-Macro ↑	TPR@1% FPR ↑	TPR@5% FPR ↑
HYDICE-Urban	0.966±0.015	0.418±0.149	0.743±0.083	0.718±0.181	0.897±0.036
Salinas	0.976±0.007	0.656±0.110	0.855±0.031	0.822±0.031	0.889±0.031
San-Diego	0.996±0.000	0.776±0.022	0.888±0.015	0.911±0.023	0.987±0.005
ABU-Airport	0.940±0.008	0.375±0.044	0.724±0.010	0.473±0.052	0.800±0.007
ABU-Beach	0.972±0.005	0.430±0.034	0.716±0.020	0.599±0.092	0.806±0.057
ABU-Urban	0.984±0.006	0.653±0.115	0.834±0.045	0.729±0.106	0.926±0.034
Arizona	0.538±0.018	0.226±0.031	0.650±0.014	0.246±0.019	0.276±0.017
Average	0.910	0.505	0.773	0.643	0.797

**Table 7:** Effect of optional drift-aware test-time adaptation (DTA) under all-domain training and LODO evaluation. Frozen and DTA rows report the mean ± standard deviation of the dataset-macro value across three paired runs. Δ is the mean paired change, DTA minus frozen inference.

Protocol	Mode	ROC-AUC	PR-AUC	F1	F1-Macro	TPR@1% FPR	TPR@5% FPR
All-domain	Frozen	0.938±0.006	0.623±0.039	0.648±0.012	0.822±0.006	0.705±0.031	0.838±0.027
All-domain	DTA	0.953±0.006	0.632±0.042	0.637±0.024	0.815±0.012	0.713±0.026	0.845±0.017
All-domain	Δ	+0.015	+0.009	−0.011	−0.006	+0.008	+0.007
LODO	Frozen	0.910±0.003	0.505±0.031	0.551±0.030	0.773±0.015	0.643±0.033	0.797±0.005
LODO	DTA	0.886±0.011	0.487±0.032	0.533±0.031	0.752±0.020	0.583±0.046	0.743±0.013
LODO	Δ	−0.024	−0.018	−0.019	−0.021	−0.059	−0.055

**Table 8:** Dataset-level mean paired changes from DTA. Positive values favor DTA; each value is averaged over three paired runs.

Dataset	All-Domain Δ ROC-AUC	All-Domain Δ PR-AUC	LODO Δ ROC-AUC	LODO Δ PR-AUC
HYDICE-Urban	−0.010	+0.008	−0.014	+0.129
Salinas	+0.001	+0.034	−0.003	+0.058
San-Diego	+0.001	+0.050	−0.002	−0.038
ABU-Airport	−0.010	+0.008	−0.028	−0.081
ABU-Beach	+0.041	+0.186	−0.086	−0.117
ABU-Urban	−0.002	−0.065	+0.004	+0.010
Arizona	+0.081	−0.161	−0.041	−0.088

**Table 9:** ROC-AUC comparison between GMoE-AD and the six base methods. All-domain GMoE-AD uses training data from all seven datasets, whereas GMoE-AD (LODO) excludes the test dataset from training. For GMoE-AD, dataset cells report mean ± standard deviation across five all-domain runs or three LODO repetitions. Base-method values are averaged over three seeds. The final column is the dataset-macro mean; where shown, its standard deviation describes variation among the seven dataset means.

Method	HYDICE-Urban	Salinas	San-Diego	ABU-Airport	ABU-Beach	ABU-Urban	Arizona	Average
GMoE-AD (all-domain)	0.991±0.010	0.947±0.027	0.997±0.001	0.956±0.037	0.973±0.010	0.992±0.001	0.748±0.151	0.943±0.082
GMoE-AD (LODO)	0.966±0.015	0.976±0.007	0.996±0.000	0.940±0.008	0.972±0.005	0.984±0.006	0.538±0.018	0.910±0.153
Abundance	0.879	0.972	0.874	0.715	0.618	0.833	0.584	0.782±0.135
AED	0.967	0.914	0.978	0.973	0.972	0.910	0.345	0.866±0.214
FCBAD	0.995	0.807	0.963	0.788	0.955	0.722	0.555	0.826±0.146
KIFD	0.997	0.996	0.991	0.958	0.951	0.964	0.609	0.924±0.130
KRX	0.991	0.986	0.981	0.922	0.963	0.970	0.195	0.858±0.271
LSUNRSO RAD	0.998	1.000	0.975	0.737	0.981	0.766	0.338	0.828±0.226

**Table 10:** Routing-stage diagnostics computed over three fixed-seed evaluations of the reference top-2 configuration. Entropy, gate variance, and KL divergence are normalized summaries over dataset-level routing distributions; entries report ranges across the seven test datasets.

Routing Stage	Norm. Entropy	Norm. Gate Variance	KL to Uniform
Pre-top-*k* raw	0.994–0.998	0.0015–0.0050	0.0014–0.0054
Post-transform dense	0.997–0.999	0.0007–0.0023	0.0006–0.0025
Effective post-top-*k*	0.499–0.500	0.3336–0.3345	0.3486–0.5679

**Table 11:** Leave-one-base-detector-out results averaged over three seeds and seven datasets.

Removed	F1-Macro	ROC-AUC	PR-AUC	Δ ROC-AUC
None	0.8309	0.9473	0.6537	—
Abundance	0.8186	0.9369	0.6321	−0.0104
AED	0.8217	0.9450	0.6344	−0.0023
FCBAD	0.8400	0.9424	0.6665	−0.0049
KIFD	0.8110	0.9423	0.6067	−0.0050
KRX	0.8316	0.9481	0.6555	+0.0008
LSUNRSORAD	0.8288	0.9437	0.6530	−0.0035

**Table 12:** Routing-loss ablation averaged over three seeds and seven datasets.

Configuration	F1-Macro	ROC-AUC	PR-AUC	Δ ROC-AUC
Full reference	0.8231	0.9354	0.6355	—
No load balancing	0.8360	0.9426	0.6612	+0.0072
No domain diversity	0.8254	0.9457	0.6433	+0.0103
No router entropy	0.8235	0.9413	0.6407	+0.0059
No expert-logit regularization	0.8276	0.9469	0.6425	+0.0115
No routing regularization	0.8319	0.9476	0.6542	+0.0122

**Table 13:** Routing diagnostics for the full model and the two grouped routing-loss removals, averaged across seven datasets and three seeds. Maximum top-2 and minimum top-1 frequencies summarize, respectively, the most-used and least-used neural expert within each dataset.

Configuration	Norm. Entropy	Max. Gate Prob.	Max. Top-2 Freq.	Min. Top-1 Freq.
Full reference	0.8649	0.3670	0.8570	0.0284
No load balancing	0.8658	0.3884	0.9334	0.0075
No routing regularization	0.9950	0.2698	0.8726	0.0141

**Table 14:** Domain-adversarial ablation averaged across all datasets. The two variants remove either the domain-classifier head or the Gradient Reversal Layer (GRL) from the full GMoE-AD configuration.

Training	Precision	Recall	F1-Macro	ROC-AUC
Full GMoE-AD	0.840	0.843	0.821	0.943
No domain classifier head	0.729	0.782	0.753	0.918
No GRL	0.742	0.783	0.761	0.924

## Data Availability

The six public hyperspectral datasets analyzed in this study are available from the repositories cited in Section 3.11. The Arizona dataset and the source code supporting the reported results are available from the corresponding author upon reasonable request, subject to applicable data-sharing restrictions.
